# Metabolomic characterisation of the glioblastoma invasive margin reveals a region-specific signature

**DOI:** 10.1016/j.heliyon.2024.e41309

**Published:** 2024-12-21

**Authors:** James Wood, Stuart J. Smith, Marcos Castellanos-Uribe, Anbarasu Lourdusamy, Sean T. May, David A. Barrett, Richard G. Grundy, Dong-Hyun Kim, Ruman Rahman

**Affiliations:** a Children's Brain Tumour Research Centre, School of Medicine, Biodiscovery Institute, University of Nottingham, UK; b Nottingham Arabidopsis Stock Centre, School of Biosciences, University of Nottingham, UK; c Centre for Analytical Bioscience, Advanced Materials and Healthcare Technologies Division, School of Pharmacy, University of Nottingham, UK

**Keywords:** Glioblastoma, Metabolomics, Invasive margin, Liquid-chromatography mass spectrometry

## Abstract

Isocitrate dehydrogenase wild-type glioblastoma (GBM) is characterised by a heterogeneous genetic landscape resulting from dynamic competition between tumour subclones to survive selective pressures. Improvements in metabolite identification and metabolome coverage have led to increased interest in clinically relevant applications of metabolomics. Here, we use liquid chromatography–mass spectrometry and gene expression microarray to profile integrated intratumour metabolic heterogeneity, as a direct functional readout of adaptive responses of subclones to the tumour microenvironment. Multi-region surgical sampling was performed on five adult GBM patients based on pre-operative brain imaging and fluorescence-guided surgery. Polar and hydrophobic metabolites extracted from tumour fragments were assessed, followed by putative assignment of metabolite identifications based on retention times and molecular mass. Class discrimination between tumour regions through showed clear separation of tumour regions based on polar metabolite profiles. Metabolic pathway assignments revealed several significantly altered metabolites between the tumour core and invasive region to be associated with purine and pyrimidine metabolism. This proof-of-principle study assesses intratumour heterogeneity through mass spectrometry-based metabolite profiling of multi-region biopsies. Bioinformatic interpretation of the GBM metabolome has highlighted the invasive region to be biologically distinct compared to tumour core and revealed putative drug-targetable metabolic pathways associated with purine and pyrimidine metabolism.

## Highlights

• Glioblastoma invasive margin harbours distinct metabolome to proliferative tumour core.
• Putative drug-targetable metabolic pathways associated with purine and pyrimidine metabolism.
• Invasive margin neuronal signatures hampers tumour discrimination.

## 1 Introduction

Isocitrate dehydrogenase wild-type (IDH-WT) glioblastoma (GBM) is the most common adult malignant neoplasm (46.1 %) of the central nervous system (CNS). Despite advances in cancer therapy and management, only 26.2 % of adults survive 5 years after diagnosis [1]. In considerable part, this is due to intra-tumour heterogeneity (ITH), which describes the genetic heterogeneity arising from a hierarchy of multiple competing subclones that dynamically change in response to therapy or pressures within the tumour microenvironment. The identification of clonal subpopulations that express different RTKs (EGFR, PDGFRA, and MET) in a mutually exclusive manner fuelled interest into characterising intra-tumour heterogeneity in GBM [2,3]. Spatiotemporal evaluation of ITH in GBM was conducted by Sottoriva et al. (2013) in a novel surgical procedure utilising the fluorescent compound 5-aminolevulinic acid (5-ALA) to aid multi-region sampling of the tumour mass in real-time. Analysis of genome-wide copy number aberrations (CNAs) revealed heterogeneity in putative GBM drivers including gain/amplification of *PDGFRA*, *MDM4*, and *AKT3*, and deletion of *PTEN*. Reconstruction of tumour fragment phylogenies to infer GBM evolution based on common, shared, and unique genetic events, identified CNAs in *EGFR* and *CDKN2A/B/p1*4ARF as early trunk events followed by amplification of *PDGFRA* and deletion of *PTEN* as subsequent branching events [4]. A similar sampling strategy coupled with RNA-sequencing of contrast-enhancing (CE) core and non-enhancing (NE) margins of GBM tumours indicated that CE regions were of the Proneural, Classical, or Mesenchymal subtype, whereas the NE regions were predominantly of the neural subtype [5]. Even more extensive characterisation of GBM ITH has been conducted at the single-cell level through RNA-sequencing, revealing a stemness gradient that was differentially associated with Verhaak expression signatures, with the more stem-like cells of Proneural and Classical subtype [6].

Since several signalling pathways converge on a limited number of metabolic pathways, targeting tumour metabolism may represent a way of circumventing the inherent genetic redundancy evident within cells and between clonal subpopulations that accounts for the failure of targeted therapies over prolonged use. Despite only detecting a limited coverage of the metabolome, ^1^H nuclear magnetic resonance (NMR) spectroscopy can detect distinct metabolic profiles from several CNS tumours, including GBM [7,8,9,10]. Early evidence of intra-tumour metabolic heterogeneity was provided by Li and colleagues who identified voxels with different values of several metabolic indices based on Cho, Cr, NAA, lactate, and lipid levels [11].

^1^H NMR has also been used to profile lipids and cholesterol species in gliomas. Compared to normal brain, gliomas demonstrated higher total cholesterol content (cholesterol esters and free cholesterol) and triglyceride levels [12]. Tugnoli and colleagues attributed the presence of cholesterol esters and triglycerides within glioma tissue to neovascularisation processes due to a positive correlation with lipid extracts taken from human blood. Interestingly, GBMs featured higher quantities of choline-containing phospholipids, including phosphatidylcholine and sphingomyelin, compared to normal brain tissue [13], likely reflecting changes to membrane structure due to aberrant biosynthetic and catabolic reactions. These studies demonstrate the versatility of ^1^H NMR for classification purposes and as a tool to probe tumour metabolism, particularly when stable isotopic substrates are utilised. However, the drawback to this technique is the poor coverage of the metabolome and lipidome which limits extensive evaluation of tumour metabolism. More recently, an elegant study integrated liquid chromatography-mass spectrometry (LC-MS)-based metabolomic data with transcriptomics, to reveal the conservation of GBM metabolic programs despite divergent molecular pathways driving tumour progression and aggressiveness. Such programs likely contribute to enhanced metabolic heterotrophy (co-opting mechanisms to actively import diverse macromolecules from the microenvironment), supporting survival within distinct ecological tumour niches and representing a metabolic hallmark of cancer [14].

Given the genetic basis underlying certain metabolic phenotypes, we hypothesised that ITH in GBM would manifest at the level of metabolism. Evidence of such a phenomenon has previously been highlighted in lung [15] and kidney [16] cancers. The identification of intra-tumour metabolic heterogeneity in GBM would have major implications in the clinic since targeting of metabolism using single agents may have the same drawbacks as previously tested targeted agents, such as RTK inhibitors, in which redundant signalling pathways or sub clonal selection allows therapies to be circumvented and resistance to develop. Understanding the genetic basis of such heterogeneity is an important means to predict metabolic phenotypes based on a genetic signature and enable patient-tailored therapeutic strategies in the clinic. We therefore aimed to characterise the level of intra-tumour metabolic heterogeneity in GBM using LC-MS, followed by integration with transcriptomics data to better understand gene transcription-metabolism correlations within surgically derived clinical tissue.

## 2 Materials and methods

### 2.1 Patient tissue collection

Patient recruitment was conducted within UK National Health Service (NHS) Trust centres at Nottingham Queen's Medical Centre, Leicester Royal Infirmary, and Royal Derby Hospital. The study was ethically approved by the National Research Ethics Service Committee for the East Midlands, UK. All research involving patient-derived tumour tissue was performed in accordance with relevant UK NHS Trust guidelines/regulations. Informed verbal consent was obtained by the operating neurosurgeon (SS) before surgery in accordance with the Code of Practice for Research issued by the Human Tissue Authority. Multi-region sampling with or without 5-ALA administration was conducted on patients with suspected GBM based on clinical history and magnetic resonance imaging (MRI) sequences, including T1-and T2-weighted (Supplementary Table 1) and intra-tumour regional classifications confirmed intraoperatively (Supplementary Table 2). The age range of patients was 33–54 years of age, with a 3:2 female:male ratio.

### 2.2 Tumour tissue sample handling

Tissue surplus to pathological confirmation of diagnosis at surgery, was immediately frozen in liquid nitrogen and stored at −80ᵒC. Tumour tissue obtained from patients was initially divided into fragments using a scalpel. The cutting surface was in direct contact with a bed of dry ice to prevent tissue from thawing, a process which would allow enzymatic reactions to occur and alter/degrade metabolites, lipids, or RNA. The wet weight of each tumour fragment was measured prior to metabolite or RNA extraction.

### 2.3 Tumour tissue preparation for LC-MS

Patient tissue weighing between 10 and 39 mg was homogenised for methanol/chloroform extraction of metabolites and lipids. Disruption of tissue in 100 μL of ice-cold (4ᵒC) methanol (MeOH) of high-performance liquid chromatography (HPLC)-grade was performed using a handheld homogeniser (Bibby Scientific Stuart; SHM1). Three hundred μL of ice-cold HPLC-grade chloroform was then added to each sample, vortexed and repeated with 100 μL ice-cold HPLC-grade water. Samples were then centrifugated for 10 min at 13000×*g* at 4ᵒC to separate the two extraction phases (upper phase containing polar metabolites and lower phase containing non-polar compounds including lipids). Lower phases were dried using a vacuum evaporator (SpeedVac; ThermoFisher Scientific) set to room temperature, followed by reconstitution in 100 μL isopropanol. Samples were then centrifugated for 10 min at 13,000×*g* at a temperature of 4ᵒC. Supernatants were carefully removed and stored in MS vials at −80ᵒC. Prior to LC-MS analysis, MeOH/H_2_O extracts were transferred into MS vials and quality controls (QCs) were prepared by pooling 5 μL from each sample.

### 2.4 LC-MS

Metabolites in the upper phase were separated using a zwitterionic-hydrophilic interaction liquid chromatography (ZIC-pHILIC) column (150 × 4.6 mm^2^, 5 μm) maintained at 45 °C in a ThermoFisher Dionex UltiMate 3000 LC system (Thermo Fisher Scientific, UK). A linear LC gradient from 80 % B to 5 % B was used over 15 min followed by a 5 min wash with 5 % B and 7 min re-equilibration with 80 % B at a flow rate of 300 μL/min, where B was 100 % acetonitrile and A was 20 mM ammonium carbonate in 18.2 MΩ water (Elga Maxima; Elga LabWater). Injection volume was set to 10 μL and samples were maintained at 4 °C. MS was performed using an Orbitrap Exactive (Thermo Fisher Scientific, UK) with a HESI-II probe operated in a polarity switching mode and using the following settings: resolution 50,000, AGC target: balanced, *m/z* range 70–1,400, sheath gas 40, auxiliary gas 5, sweep gas 1, probe temperature 150 °C, and capillary temperature 275 °C. For positive mode ionisation: source voltage +4.5 kV, capillary voltage +40 V, tube voltage +70 V, skimmer voltage +20 V. For negative mode ionisation: source voltage −3.5 kV, capillary voltage −30 kV, tube voltage −70 kV, skimmer voltage −18 kV. Mass calibration was performed before each batch. The mass range was extended to cover small metabolites by inclusion of low-mass ions to the standard Thermo calmix masses (below *m*/*z* 1400), C_2_H_6_NO_2_ (*m*/*z* 76.0393) for ESI+ and C_3_H_5_O_3_ (*m*/*z* 89.0244) for ESI-. Number of technical replicates used were n = 3 to n = 6 per tumour region, depending on availability of tissue obtained from surgery.

Lipids extracts from patient tumour tissue was initially separated using a reverse phase ACE Excel 2C18 column 50 × 2.1 mm column [17] equipped with an appropriate guard column (ThermoFisher Scientific, UK) to act as a filter preventing particulates from entering the reverse phase column and held at 50ᵒC in an Acella modular system (ThermoFisher Scientific, UK) with cooled autosampler, column oven and quaternary pumps. The injection volume was set to 10 μL, with samples held at 10ᵒC. LC mobile phases consisted of A [60 % of 0.1 % MS-grade ammonium acetate (final volume) in 18.2 MΩ water (Elga Maxima; Elga LabWater) with 40 % acetonitrile] and B [10 % of 0.1 % MS-grade ammonium acetate (final concentration) in 18.2 MΩ water (Elga Maxima; Elga LabWater) with 10 % acetonitrile and 80 % isopropanol (MS-grade, VWR, UK]. MS was performed using an Exactive Orbital ion-trap mass spectrometer (ThermoFisher Scientific, UK) acquiring data simultaneously in full scan ion mode (*m*/*z* 100–1900; resolution 25,000) in both positive and negative polarity switching modes. The flow rates of sheath gas, desolvation gas and sweep gas were 30, 15 and 5 units, respectively. The capillary and desolvation heater temperatures were set to 250ᵒC and 300ᵒC, respectively. The spray voltage was set to 4000 V. Local control of the LC system was conducted using Xcalibre 2.0.7, which was also used for MS control and data acquisition.

### 2.5 Metabolomic and lipidomic data analysis

Raw LC–MS data were processed with XCMS for untargeted peak-picking [18] and mzMatch for alignment and annotation of related peaks [19]. IDEOM software was used for noise filtering and putative metabolite identification, as shown previously [20]. Univariate analysis using one-way ANOVA followed by a post hoc comparison using a two-sided *t*-test to select differentially abundant mass ions between tumour regions was computed. The variances were not assumed to be equal between each tumour region, and the adjusted p-value cut-off threshold was set 0.05 following correction for multiple testing problem using false discovery rate (FDR). The mass ions were identified using IDEOM with default parameters whereby retention time for the identification of authentic standards was set 5 %, retention time for the identification of calculated retention time was set to 50 %, and mass accuracy for mass identification was set to 5 ppm. Putative metabolites were also identified using the Human Metabolome Database (HMDB). Generally, metabolite identification was performed by matching accurate masses and retention times of authentic standards Level 1 metabolite identification according to the metabolomics standards initiative [21,22], but when standards were not available, predicted retention times were employed, hence these identifications should be considered as putative (Level 2 identification). Specifically, differentially abundant metabolites were identified by matching masses and retention times to an existing database with a mass accuracy window of 3 ppm. Additional automated noise and MS artifact filtering procedures were applied to remove peak sets which either contained peaks present at equal or higher abundance in blank solvent samples, peaks lower than the intensity threshold (10,000), shoulder peaks or duplicate peaks within the same mass (3 ppm) and retention time (0.2min) window, and common MS artifacts.

Database searches for lipid was conducted using HMDB, Lipid Maps and Metlin at an accuracy of 5 parts per million (ppm). LC-MS chromatograms were viewed and displayed using Xcalibre 3.0.63. Metabolomics QC and data analysis was performed using the free online MetaboAnalyst 4.0 tool [23]. Using either the metabolite set enrichment analysis (MSEA) or pathway analysis suite, metabolites with non-human putative identifications were removed from the set of variables. Data analysis was then performed according to the protocol outlined by Ref. [24]. Briefly, peak intensity values for each identified metabolite were uploaded followed by the removable of variables with >50 % missing values and imputation of remaining zero values with half of the minimum positive value. Filters were then applied to remove features if their relative standard deviations (RSDs) were >30 % in the QC samples. Sample normalisation based on total ion count was performed using IDEOM software. Therefore, only log transformation of the data sets was applied. For univariate analysis, no data scaling was applied, whereas Pareto scaling was applied prior to multivariate analysis. Other features of MetaboAnalyst 4.0 were used to generate heatmaps and dendrograms, featuring hierarchical clustering of samples and metabolites, as well as principal component analysis (PCA) and correlation analysis. Lists of metabolites identified as significant were compared against pathway-associated metabolite sets within the MSEA suite or analysed for pathway impact within the pathway analysis suite of MetaboAnalyst. Orthogonal partial least squares-discriminant analysis (OPLS-DA) of metabolomics and lipidomic data was conducted using Umetrics SIMCA-P 13 software.

### 2.6 Transcriptomics

RNA extraction from tumour tissue was performed using the mirVana miRNA isolation kit (ThermoFisher Scientific; AM1561), whereby total RNA was extracted as per the manufacturer's protocol and RNA eluted with 100 μl nuclease-free water at 95 °C, with no further enrichment for microRNAs. DNase treatment of RNA samples was performed using the TURBO DNA-free kit (ThermoFisher Scientific; AM1907). Initial quality assessment of the RNA samples was performed using an Agilent RNA 6000 Nano kit and run on an Agilent Bioanalyzer 2100 to generate RNA Integrity Numbers. Transcriptomics was conducted on 1000 ng of RNA (100 ng/μL) using Affymetrix Human Gene ST2.1 Strips and using three replicates per sample. The Affymetrix Human Gene 2.1 ST 16-Array provides an accurate, sensitive, and comprehensive measurement of protein coding and long intergenic non-coding RNA transcripts, with >30,000 coding transcripts. Briefly, the Affymetrix GeneChip WT PLUS Reagent Kit was used to create double-stranded cDNA from the RNA template, from which cRNA was produced as a template for a second round of cDNA synthesis. The resultant single-stranded cDNA library was fragmented, terminally labelled, and hybridised to the strips using the Affymetric GeneAtlas Hybridisation, Wash, and Stain Kit for WT Array Strips. Prior to downstream differential expression analysis, evaluation of array hybridisation was conducted using the Partek® Genomics Suite to identify artifacts on the chip that would generate spurious results.

### 2.7 Transcriptomics analyses

Multilevel linear modelling and differentially expressed gene (DEG) analysis was conducted using R software. The limma package uses a multilevel linear modelling approach to analyse microarray data. The approach requires two matrices to be specified; the first is the design matrix which indicates in effect which RNA samples have been applied to each array; the second is the contrast matrix which specifies which comparisons are to be made between the RNA samples. A linear model is fitted to the data which fully models the systematic part of the data. The model is specified by the design matrix. Each row of the design matrix corresponds to an array in the experiment, and each column corresponds to a coefficient that is used to describe the RNA sources. Briefly, raw data was pre-processed with the *oligo* package [25] to mediate quantile normalisation using the Robust Multi-array Average (RMA) method. Assessment of two QC metrics of the data was performed by visualising Relative Log Expression (RLE) [compares the expression level of one probe set against the median expression of the same probe set across samples] and Normalised Unscaled Standard Errors (NUSE) [standardises standard error estimates across arrays so that the median standard error for the probe set is 1 across all arrays]. Low variance genes were then removed using the *genefilter* package [26] prior to differential gene expression analysis using the *limma* package [23]. The significance of differentially expressed genes (DEGs) was determined using a 0.05 p-value cut-off after correction for multiple comparisons using the Benjamini-Hochberg (BH) method. Gene ontology (GO) enrichment analysis of DEGs was conducted using the *topGO* package by applying statistical tests based on either gene counts (Fisher's exact test) or gene scores (Kolmogorov-Smirnov-like test) to different algorithms. The ‘classic’ algorithm tests each GO term independently, therefore not taking the GO hierarchy into account. The ‘weight01’ algorithm is a mixture of the ‘elim’ [traverses the GO hierarchy from bottom to top, discarding any genes that annotated with significantly enriched descendant GO terms] and ‘weight’ [connected nodes are compared to detect the most locally significant GO terms] algorithms introduced by Ref. [27].

### 2.8 Statistical analyses

For metabolomics, univariate models were conducted with a *p*-value cut-off of 0.05 following corrections for multiple comparison based on FDR. In two-sample *t*-test comparisons, variances were not assumed to be equal between groups. For transcriptomics, the significance of differentially expressed genes (DEGs) was determined using a 0.05 *p*-value cut-off after correction for multiple comparisons using the Benjamini-Hochberg (BH) method. Lists of DEGs along with log2 expression values were inputted into the NetworkAnalyst online interface and assessed using either of the curated databases IMEx [28] or String [29] with a confidence score cut-off of 900.

Multi-omics data integration of metabolites and transcripts was performed through pathway-based network analysis using Cytoscape (v.3.4.0) with MetScape 3 (v.3.1.3) plugin, applying a minimum fold change threshold of 1 and a p-value <0.05.

## 3 Results

### 3.1 GBM intratumor metabolic heterogeneity

**Fig. 1:**
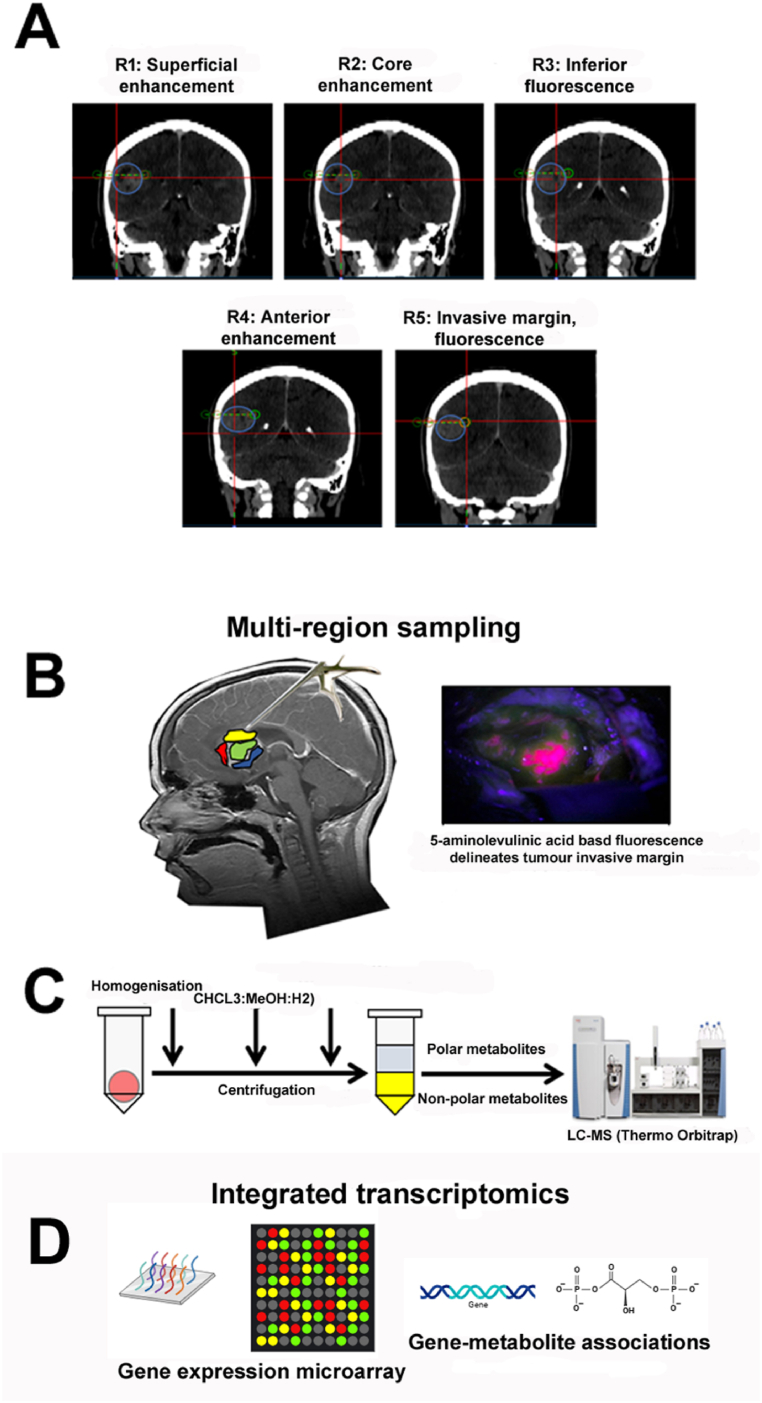
**GBM intratumour methodology pipeline incorporating stereotactic neuronavigation and integrated metabolic and transcriptomic profiling.** (A) Stereotactic CT images of patient 9 are representative of the use of StealthStation™ neuro-navigation tool. The outline of the tumour mass is indicated by the blue circle. White areas indicating the tumour mass represent areas of blood-brain barrier breakdown, with the true edge of the tumour extending beyond the contrasted area but undefinable. Target selection was conducted utilising information from a series of MRI sequences. Region (symbol R) designations are labelled within the figure. Locations of each region are depicted by the red cursor. The green dotted line denotes the scope probe used to identify regions of interest. (B) Schematic representation of multiple intratumour region sampling (4–5 per patient) during standard surgical treatment (left). Administration of 5-aminolevulinic acid (5-ALA) assists surgical excision of minimal disease beyond the main tumour mass (right). (C) Biphasic extraction of both polar and non-polar metabolites from each tumour fragment for LC-MS-based metabolomic and lipidomic analyses. (D) RNA extracted from same tumour regions, analysed via gene expression microarray to enable integrated transcriptomics and identification of gene-metabolite associations.

We performed LC-MS on GBM intra-tumour fragments taken from four to five different regions selected by pre- and intra-operative neuro-navigation using StealthStation™ (Fig. 1A) and by 5-ALA-based isolation of invasive margin tissue (Fig. 1B) (for clinical information and sample regional information respectively, see Supplementary Tables 1 and 2). This surgical sampling scheme aimed to determine if the heterogeneity observed at the genomic level manifests phenotypically as intra-tumour metabolic heterogeneity within the global metabolite (metabolome) and lipid (lipidome) spheres (Fig. 1C), and furthermore, whether integrated transcriptomic profiling identifies key underpinning gene-metabolite associations (Fig. 1D).

Staining of tumour fragments using haematoxylin and eosin (Supplementary Fig. 1) or Ki67 (Supplementary Fig. 2) confirmed the heterogenous nature of GBM at a cellular level regarding density, morphology, and proliferative potential. Mass spectrometric analysis of tumour fragments generated a spatial and quantitative output that was used to test the following hypotheses: 1) GBMs harbour a heterogeneous metabolic profile featuring niches delineated by distinct metabolomic and lipidomic profiles; 2) The therapy-relevant invasive margin is characterised by a distinct metabolomic and lipidomic signature that may be amenable to metabolism-based therapeutic intervention; 3) Metabolic and lipid profiles functionally represent underlying genomic heterogeneity in metabolism-related genes.

Multi-region sampling (4–5 regions per patient) was conducted on five patients with pathology-confirmed diagnoses of high-grade glioma (HGG). Four patients (6, 8, 9, and 15) had typical GBMs whereas one patient (14) had a malignant glioneuronal tumour (MGNT), a high-grade glioma with neuronal characteristics. Of the four GBMs, three were identified as *IDH1* wildtype (6, 8, and 9), whereas patient 15 expressed the mutant R132H variant (Supplementary Table 1). Prior to surgery, patients 6, 9 and 14 were administered 5-ALA to facilitate complete resection of residual disease within the invasive margin, residing in close proximity to micro deposits of neoplastic cells likely to initiate the tumour recurrence.

### 3.2 LC-MS based profiling of HGG metabolism

To initially explore between-patient variance, one-way ANOVA and metabolite set enrichment analyses was used to identify metabolites significantly variant commonly across all five patients (Table 1, Table 2).

**Table 1:** **List of metabolites commonly identified across all five patients as significantly variant using a one-way ANOVA model.** ID confidence scores delineate the confidence of the putative metabolite identification, where 10 = standard retention time within 5 %, in preferred database (Human Metabolome Database), 9 = standard retention time within 5 %, not in preferred database, 8 = standard retention time within 5 %, in preferred database and related peak (mz match), 7 = standard retention time within 5 %, not in preferred database and related peak (mz match), 6 = calculated retention time within 50 %, in preferred database and related peak (mz match), 5 = calculated retention time within 50 %, not in preferred database and related peak (mz match).

Metabolite	ID confidence
Alpha-ketoisovaleric acid	10
L-Ornithine	10
Glycerophosphocholine	10
N-Acetylglutamine	9
(S)-2,3,4,5-Tetrahydropyridine-2-carboxylate	8
2-Dehydro-3-deoxy-L-rhamnonate	8
3-Sulfinoalanine	8
4-Guanidinobutanoic acid	8
Anserine	8
Carnosine	8
D-Glucose	8
D-Proline	8
N-Acetyl-L-aspartate	8
(S)-3-Methyl-2-oxopentanoic acid	8
1-(beta-D-Ribofuranosyl)-1,4-dihydronicotinamide	7
Asn-Lys-Asn-Pro	7
Cystenyl-Serine	7
Glutamylleucine	7
Glu-Phe-Asn-Arg	7
Leucyl-Lysine	7
Leucyl-Threonine	7
Maleamate	7
Met-Ala-Ser	7
Met-Gly-Ser	7
N-Acetylaspartylglutamate	7
4-Pyridoxic acid	6
L-Methionine S-oxide	6
1-Pyrroline-5-carboxylate	6
Glycerophophoethanolamine	6
2-Octenoylcarnitine	5
Ala-Ala-Ala	5
Asp-Thr-Thr-Asp	5
Glu-Thr-Thr	5
Methionyl-Asparatate	5

**Table 2:** Metabolite set enrichment analysis of metabolites in all patients identified as significant using one-way ANOVA.

Patient 6:						
Metabolite Set	Total	Expected	Hits	Raw p	Holm p	FDR
Glucose-Alanine Cycle	13	1.56	5	0.0134	1	1
Histidine Metabolism	43	5.17	9	0.0623	1	1
Glycine and Serine Metabolism	59	7.09	11	0.0847	1	1
Malate-Aspartate Shuttle	10	1.2	3	0.108	1	1
Carnitine Synthesis	22	2.64	5	0.113	1	1

Patient 8:						
Metabolite Set	Total	Expected	Hits	Raw p	Holm p	FDR

Glutamate Metabolism	49	13.7	19	0.0631	1	1
Phosphatidylethanolamine Biosynthesis	12	3.36	6	0.0878	1	1
Aspartate Metabolism	35	9.81	13	0.152	1	1
Urea Cycle	29	8.13	11	0.16	1	1
Cardiolipin Biosynthesis	11	3.08	5	0.168	1	1

Patient 9:						
Metabolite Set	Total	Expected	Hits	Raw p	Holm p	FDR

Phenylacetate Metabolism	9	0.729	3	0.0302	1	1
Glycolysis	25	2.03	5	0.0452	1	1
Arginine and Proline Metabolism	53	4.3	8	0.0574	1	1
Glutathione Metabolism	21	1.7	4	0.083	1	1
Glutamate Metabolism	49	3.97	7	0.0936	1	1

Patient 14:						
Metabolite Set	Total	Expected	Hits	Raw p	Holm p	FDR

Phosphatidylethanolamine Biosynthesis	12	3.33	7	0.0246	1	0.989
Cardiolipin Biosynthesis	11	3.05	6	0.0543	1	0.989
Phosphatidylcholine Biosynthesis	14	3.88	7	0.0629	1	0.989
Argine and Proline Metabolism	53	14.7	20	0.068	1	0.989
De Novo Triacylglycerol Biosynthesis	9	2.5	5	0.0725	1	0.989

Patient 15:						
Metabolite Set	Total	Expected	Hits	Raw p	Holm p	FDR

Phosphatidylethanolamine Biosynthesis	12	2.92	7	0.0117	1	0.789
Cardiolipin Biosynthesis	11	2.67	6	0.0294	1	0.789
Pyrimidine Metabolism	59	14.3	21	0.0305	1	0.789
Phosphatidylcholine Biosynthesis	14	3.4	7	0.0322	1	0.789
Argine and Proline Metabolism	53	12.9	18	0.0681	1	0.789

**Fig. 2:**
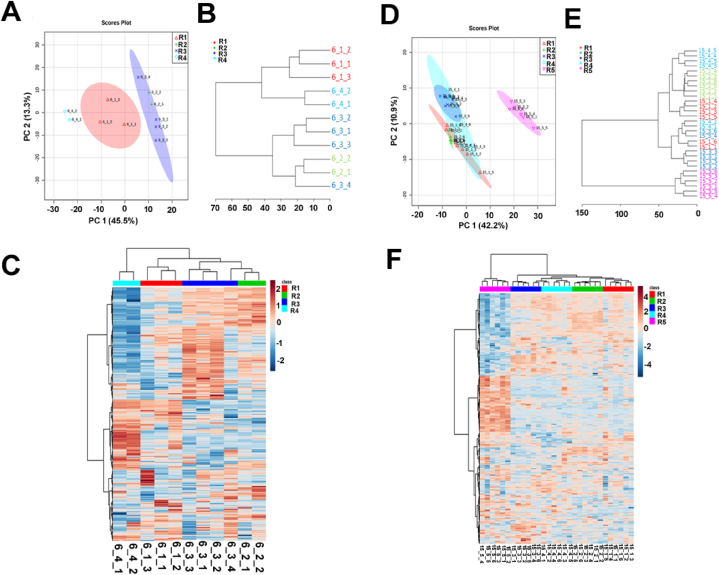
**Metabolomic plots for representative patients**. *(A-C) Patient 6, (D-F) Patient 15*. (A, C) Dimensional reduction through PCA and visualization of sample variation. (B, D) Hierarchical clustering analysis measuring dissimilarity between samples. (C, F) Heatmap overview of the metabolome. Sample IDs delineate the patient, region (symbol R), and replicate numbers, respectively. The colour scale for heat intensity is shows normalised peak intensity values of metabolites, where red indicates a significant increase, and blue represents a significant decrease.

We first selected patient 6 and 15 for paired inter-tumour metabolomic comparisons (Supplementary Tables 3 and 4), representative of *IDH* wild type and R132H variant GBM respectively. Raw LC-MS data was pre-processed by peak identification, alignment, annotation, noise filtering, and putative identification of metabolites. Peak intensities were then normalised to total ion count to account for differences in tissue weight and metabolite content. PCA of metabolomic profiles revealed regional heterogeneity as well as overlap between regions. In patient 6, the first principal component largely separated each tumour region, accounting for 45.5 % of the variation in the data (Fig. 2A). The horizontal plane indicated that regions 6_2 and 6_3 are more similar in comparison to regions 6_1 and 6_4, as supported by hierarchical clustering of the samples (Fig. 2B). Clustering of the samples also demonstrated a shorter distance measure between replicate 4 of region 6_3 (designated as 6_3_4) and fragments from region 6.2, supported by metabolomic similarities as depicted in the heatmap overview. Since these regions were located adjacent to each other within the original tumour mass, it is possible that fragment 6_3_4 was derived from the same metabolic niche containing region 6_2 (Fig. 2C). In patient 15, separation of samples along the first component demonstrated that most of the variation occurred between region 15_5 and all other regions collectively (Fig. 2D), supported by low dissimilarity between regions 15_1, 15_2, 15_3 and 15_4 and clustering of fragments from these regions (Fig. 2E). The heatmap overview of patient 15's metabolome (Fig. 2F) highlighted a largely homogenous metabolome within the non-invasive regions compared to patient 6. Both patients showed separation and clustering of the invasive margin from non-invasive regions in support of our hypothesis that the invasive margin displays a distinct metabolomic signature.

We next conducted metabolomic profiling of patients 8, 9 and 14. PCA analysis of patient 8 highlighted clustering of samples from each region and emphasised the unique metabolome demonstrated by region 8_2 (Supplementary Fig. 3). region 8_3 demonstrated increased levels of several amino acids relative to other regions, including glycine and l-glutamate. Comparison of non-invasive and invasive regions revealed a general decease in essential and conditional amino acids that reached significance in most cases Supplementary Table 5). PCA analysis of patient 9 highlighted overlapping metabolomic signatures between regions 9_2, 9_3, 9_4 and 9_5, and where Region 9_1 demonstrated a distinct metabolome as supported by hierarchical analysis (Supplementary Fig. 4). PCA analysis of patient 14 demonstrated overlap between regions 14_1, 14_2 and 14_3 with separation of regions 14_4 and 14_5 from the main cluster as reflected through hierarchical analysis (Supplementary Fig. 5). The invasive region in patients 9 and 14 both demonstrated increased levels of early glycolytic intermediates, but this was associated with decreased levels of ATP and NADH compared to non-invasive regions (Supplementary Tables 6 and 7).

### 3.3 Metabolites differ in extent of heterogeneity across HGG regions

**Fig. 3:**
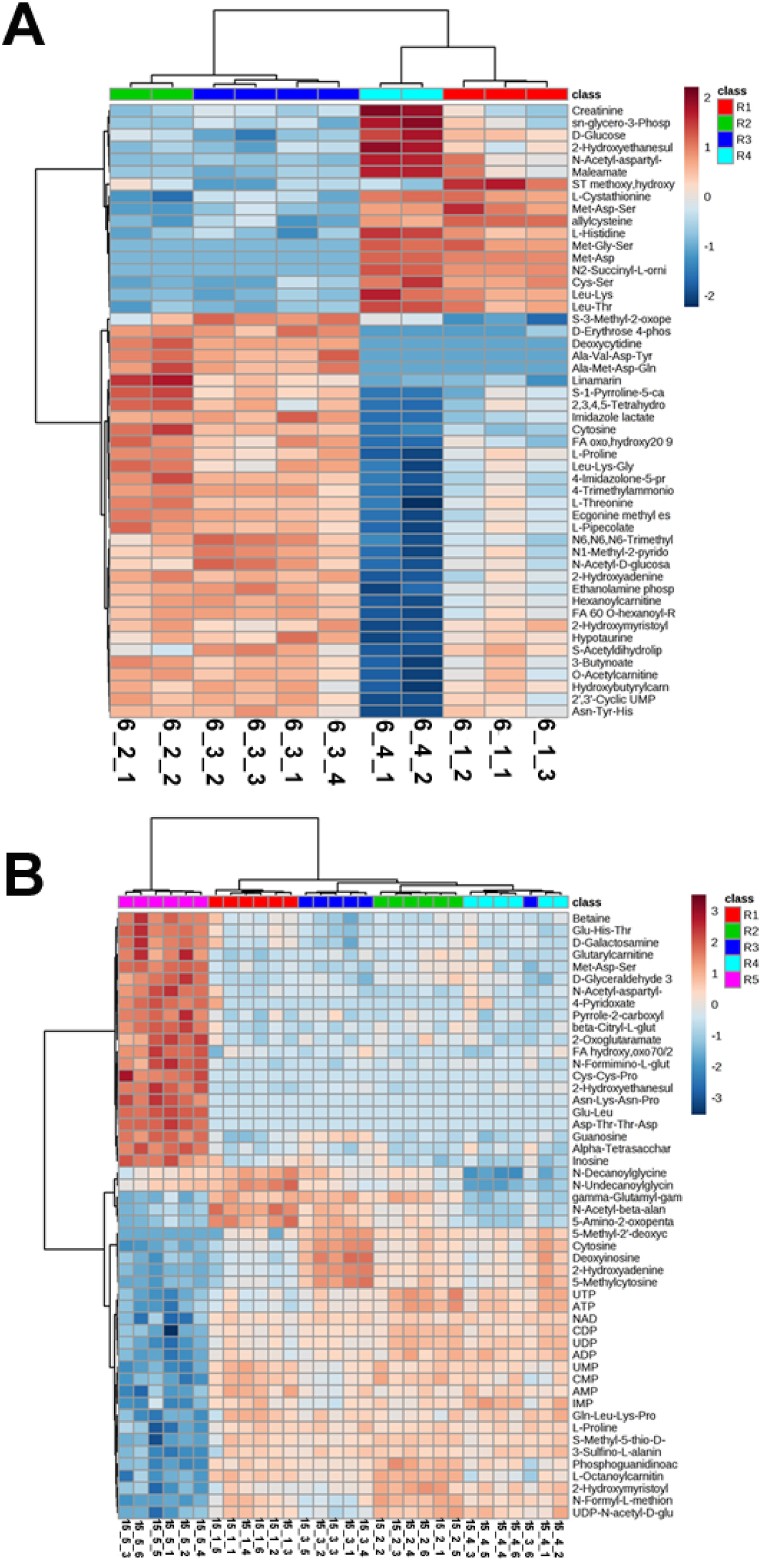
**Identification of regionally variant metabolites in representative patients**. One-way ANOVA was conducted on the metabolomic profiles from (A) patient 6 and (B) patient 15. Only the top 50 significant metabolites are displayed in the heatmaps. Sample IDs (bottom) delineate the patient, region (symbol R), and replicate numbers, respectively. The colour scale for heat intensity is shows normalised peak intensity values of metabolites, where red indicates a significant increase, and blue represents a significant decrease.

Multivariate analysis of the HGG metabolome revealed a more heterogeneous metabolic profile in patient 6 compared to patient 15. To determine metabolites with significant variation across regions, a one-way analysis of variance (ANOVA) test was performed to select for variables with a false discovery rate (FDR)-corrected *p*-value of less than 0.05. Heatmaps displaying the top 50 significant metabolites confirmed the findings of the multivariate analysis above, highlighting the relative similarity between regions 6_2 and 6_3 (Fig. 3A), and the stark contrast between the invasion margin and non-invasive regions of patient 15 (Fig. 3B).

**Fig. 4:**
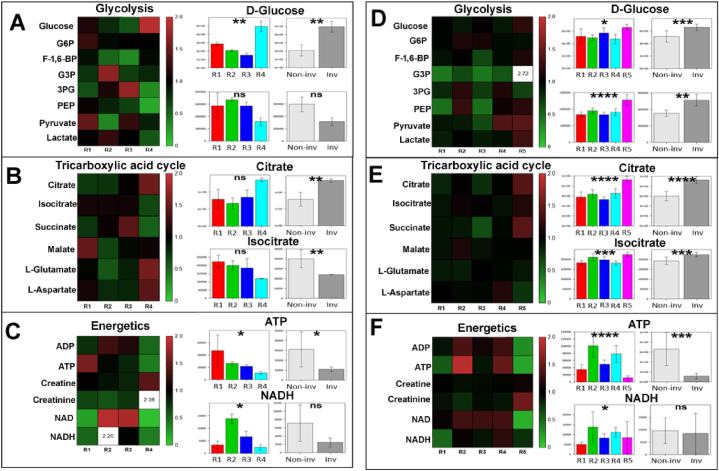
**Regional variation in metabolites generated within the glycolysis pathway, tricarboxylic acid cycle, and energetics.** (*A-C, patient 6, D-F, patient 15*) Heatmaps display median replicate values of mean-centred peak intensities for metabolites generated within glycolysis (A, D), TCA cycle (B, E), and energetics (C, F). Scale bars depict the grading of colours for fold changes ranging between 0 and 2. Fold changes above 2 are numbered. Peak intensities for metabolites of interest across regions (symbol R; left bar plot) and between region types (right bar plot) are displayed in each panel. Statistical evaluation of regional variation was conducted using a one-way ANOVA model and two-sample *t*-test, respectively. Abbreviations: Non-inv – non-invasive (light grey); Inv – invasive (dark grey). ns = not significant; ∗ = p < 0.05; ∗∗ = p < 0.01; ∗∗∗ = p < 0.001; ∗∗∗∗ = p < 0.0001.

Metabolites related to glycolysis (D-glucose, lactate), TCA cycle (citrate, isocitrate), and energetics (ATP, NADPH) exhibited regional heterogeneity in patient 6. Within the glycolysis pathway, only D-glucose had a significant ANOVA score (FDR<0.01) in reflection of regional heterogeneity and higher metabolite abundance of the metabolite within the invasive margin compared to the non-invasive regions (2.36-fold increase) (Fig. 4A). Significant scores for citrate and isocitrate were also obtained between the non-invasive regions and the invasive margin (Fig. 4B), suggestive of differences in mitochondrial activity and lipid synthesis. Regional heterogeneity was observed for several energetic species, including ATP, creatine, and NADH (Fig. 4C). Interestingly, regions 6_2 and 6_3 demonstrated an above median abundance of NAD+, as observed for its reduced equivalent NADH. Both these metabolites demonstrated a significant ANOVA score, but only NAD + levels within the invasive margin were significantly different to non-invasive regions (FDR<0.01). A similar pattern of differences confined between non-invasive and invasive regions was identified for most amino acids apart from L-glutamine, which demonstrated homogeneity across all regions that was not reflected in its immediate downstream product, L-glutamate. Although L-serine and L-glycine showed non-significant heterogeneity between regions, this did not translate into significantly variable glutathione levels (Supplementary Table 3).

Patient 15 demonstrated a relatively homogenous non-invasive metabolomic profile in terms of glycolysis (Fig. 4D) and TCA cycle (Fig. 4E). Heterogeneous levels of ADP, ATP (p < 0.0001), NAD+ and NADH (FDR<0.05) were observed in support of regional differences in energetics (Fig. 4F). Of note, several glycolytic intermediates were lower in abundance in the non-invasive regions (Fig. 4D), which may plausibly be associated with increased glycolytic metabolic flux, supported by the observed significantly increased ATP levels in non-invasive regions (Fig. 4D). Increased level of lactate in the invasive margin region is consistent with the expected conversion of pyruvate to lactate under anaerobic conditions, which corresponds to lower ATP abundance detected in invasive relative to non-invasive regions (Fig. 4F). Lactate may also enhance tumour cell motility, as seen in head and neck carcinoma cell lines, whilst reducing migration of immune cells [30]. Lactate within the invasive region may therefore be a biomarker of tumour invasion and warrants investigation in a larger cohort. This notion is supported by previous elucidation of the association between lactate efflux and GBM invasion, where the invasive phenotype was impaired upon lactate efflux inhibition and by the role of lactate dehydrogenase in promoting GBM invasion [31,32]. Levels of the L-glutamate and its precursor, L-glutamine, were relatively unchanged across all tumour regions of patient 15, suggesting a lack of anapleurotic activity (Supplementary Table 4). No significant scores for glutathione in oxidised or reduced form were observed across all regions of patient 15, suggestive of sustained synthesis to replenish metabolite pools (Supplementary Table 4). Collectively, both patients 6 and 15 highlighted the different extents of heterogeneity within several metabolic pathways that was particularly evident between the non-invasive and invasive regions.

### 3.4 Lipid heterogeneity within and between HGG patients

**Fig. 5:**
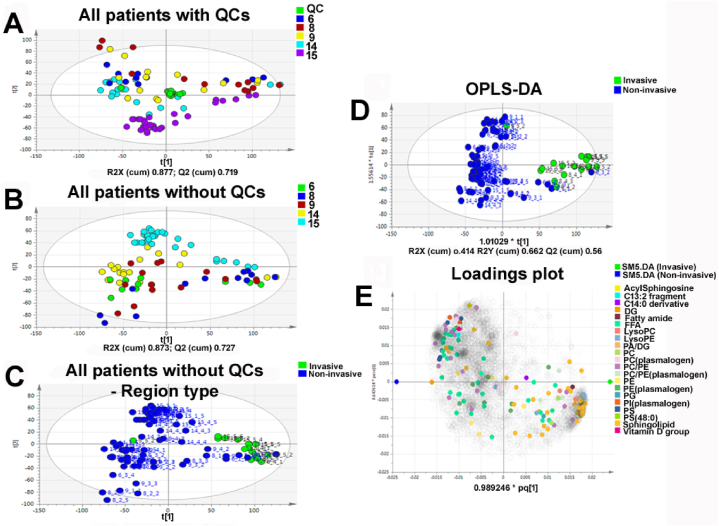
**PCA and OPLS-DA of lipidomic profiles from all five GBM patients.** (A–C) The position of tumour fragments from each patient are positioned in the model plane formed by the first two principal components in the presence (A) or absence (B and C) of the QC samples. Figure legends (top right) delineate colour choices for patient number (A and B) and region type (C). R2X (cum) and Q2 (cum) values quantitatively depict the amount of explained (goodness of fit) and predicted (goodness of prediction) variation accounted for by the fitted model, respectively. Hotelling's T2 calculated at 95 % confidence level is depicted as an ellipse within each figure. (D–E) The mass ion pairs (RT, *m*/*z*) from the lipidomic dataset were exported with their normalised abundances for multivariate analysis in which OPLS-DA was used for modelling the differences and similarities between samples. (D) The position of tumour fragments from each patient are positioned in the model plane formed by the predictive (Y) and orthogonal components (X). R2X (cum) accounts for the predictive and orthogonal variation in X. R2Y (cum) is the total sum of variation in Y explained by the model. Q2 (cum) depicts the amount of predicted (goodness of prediction) variation accounted for by the model. The loadings for each variable derived from the OPLS-DA analysis are displayed in (E). Figure legends (top right) delineate the colour choices for region type (D) and lipid species (E). Hotelling's *T*^2^ calculated at 95 % confidence level is depicted as an ellipse in (D).

The heterogeneity observed within metabolomic profiles in relation to regional variation between invasive and non-invasive regions were largely replicated within lipidomic profiles. All QC samples apart from one showed strong clustering, indicating minimal shift during the experiment duration (Fig. 5A). PCA of all patients revealed spread between tumour fragments in patients 6, 8 and 9 (Fig. 5B). Samples from patients 14 and 15 were more clustered in comparison to the other patients (Fig. 5B). ‘Clustering’ in this context indicates relative metabolomic homogeneity of intra-tumour regions from patients 14 and 15 (as determined by PCA), relative to the greater separation of intra-tumour regions from all other patients. There are thus relatively smaller differences in the measured variables (i.e., intra-tumour metabolomic profiles) within patients 14 and 15, compared to all other patients. Interestingly, patient 15 was slightly segregated from the other patients, suggestive of a distinct lipidomic signature. This is likely due to the IDH1 mutation in patient 15 influencing the lipid signature displayed by this tumour. It was evident that some samples from different tumours demonstrated a similar lipidomic profile that segregated from the main cluster of samples (Fig. 5B). Analysing the region type revealed most of these samples to be derived from the invasive margin (Fig. 5C). Sample 9_5_2 from the invasive region of patient 9 did not cluster with all other invasive region profiles and may therefore be an outlier. Collectively, this supported the notion of a highly distinct lipidomic signature within the invasive margin compared to non-invasive regions. Multivariate analysis based on class discrimination using an OPLS-DA model confirmed clustering and separation of invasive region lipidomic profiles, although some non-invasive region samples were co-segregated (Fig. 5D). The strength of the model was only moderate with R2Y = 0.662 and Q = 0.56, with R2X = 0.414. The R2X value indicates the predictive and orthogonal variation in X (data matrix) explained by the model, whereas the R2Y value indicates the total sum of variation in Y (class discrimination) explained by the model. The Q2 value depicts the goodness of prediction of the model and to identify overfitting. The most important lipid species distinguishing the invasive and non-invasive regions were sphingolipids and fatty acids, respectively, which may represent biomarkers for further evaluation. (Fig. 5E). (Kamphorst et al., 2013). However, lipid classes were identified for only a small fraction of lipids since tandem MS/MS was not conducted in the methodology. Therefore, the differences observed may not reflect the full complement of lipids classes that are altered within and between patients.

### 3.5 Multilevel modelling identifies differentially abundant metabolites between the non-invasive and invasive regions

In order to identify common changes across all patients, multilevel modelling was performed on patients 6, 8, 9, 14, and 15, covering *IDH1* wildtype GBM, *IDH1* mutant GBM, and MGNT tumour types. A multilevel model was applied since it is not statistically appropriate to treat samples from the same patient as independent.

The data confirms the significance of several metabolites associated with glycolysis, TCA cycle, energetics, amino acid synthesis and glutathione metabolism. The model emphasises the higher abundance of several amino acids, including L-proline, in the non-invasive regions compared to the invasive margin. The two metabolites with the highest fold changes, N-acetyl-L-aspartate (NAA) and N-acetyl-aspartyl-glutamate (NAAG), are neuronal markers, indicating that tissue from the invasive margin may not be solely constituted of tumour cells (Supplementary Table 8). Metabolite set enrichment analyses (MSEA) of metabolites with a fold change >1.5 or < −0.67 showed significant enrichment of *nucleotide sugars metabolism*, *starch and sucrose metabolism* and *phosphatidylcholine biosynthesis*, with respective raw *p*-values of 0.012, 0.017 and 0.031 (Supplementary Table 9). Pathway analysis of the same list of differentially abundant metabolites revealed significant impact to several pathways, including *lysine degradation*, *pyrimidine metabolism* and *arginine and proline metabolism*, with impact scores of 0.33, 0.36 and 0.32, respectively (Supplementary Table 10). However, we note the increasing evidence supporting how high-grade glioma cells functionally hijack neuronal mechanisms via synaptic cross-communications [33,34], and thereby NAA and NAAG signatures from GBM cells within invasive margin tissue cannot be excluded.

Collective analysis of differentially abundant metabolites between the non-invasive and invasive regions of patients 9, 14, and 15 demonstrated significant enrichment of several metabolic pathways. This analysis also highlighted inter-patient heterogeneity indicating that there are not only metabolites commonly altered between patients (Supplementary Table 8), but also some metabolites that are altered in some patients and not others.

### 3.6 Non-invasive regions exhibit (or are characterised by) high proline levels

**Fig. 6:**
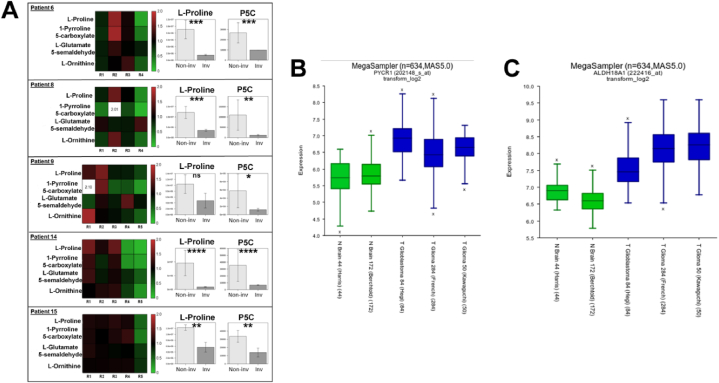
**Regional variation in all five patients of metabolites belonging to the proline metabolism pathway and expression of proline-associated genes across GBM and normal brain genomes.** (A) Heatmaps display the median replicate value of mean-centred metabolite peak intensities. Fold changes above 2 are numbered. Peak intensities for L-proline and 1-pyrroline-5-carboxylate (P5C) within the non-invasive and invasive regions are displayed in each panel. Statistical evaluation of was performed using a two-sample *t*-test, respectively. Abbreviations: Non-inv – non-invasive (light grey); Inv – invasive (dark grey). ns = not significant; ∗ = p < 0.05; ∗∗ = p < 0.01; ∗∗∗ = p < 0.001; ∗∗∗∗ = p < 0.0001. (B–C) Median expression of canonical proline pathway-associated genes pyrroline-5-carboxylate reductase 1 (PYCR1) and ALDH18A1 (encoding P5CS protein) across published GBM transcriptomic datasets in the R2:Genomics Analysis and Visualization platform. (B) Median PYCR1 expression in GBM datasets (Hegi, n = 84; French, n = 284, Kawaguchi, n = 50) of 6.93, 6.43 and 6.65 respectively, is higher than median expression (5.74 and 5.79) in normal brain (Harris, n = 44; Berchtold, n = 172) respectively. (C) Median ALDH18A1 expression in GBM datasets (Hegi, n = 84; French, n = 284, Kawaguchi, n = 50) of 7.46, 8.15 and 8.26 respectively, is higher than median expression (6.90 and 6.60) in normal brain (Harris, n = 44; Berchtold, n = 172) respectively.

These findings revealed that metabolomic heterogeneity was largely confined between non-invasive and invasive regions. Of particular interest was the observation of consistently higher levels of L-proline within non-invasive regions compared to invasive margin in all five patients (Fig. 6A). L-glutamate 5-semialdehyde (GSA), another product of proline catabolism, showed a positive correlation with L-proline in patients 8, 14 and 15 (p < 0.01, p < 0.0001, p < 0.0001 respectively). Transfer of the amino group from L-glutamate to GSA yields L-ornithine, which is part of the urea cycle. In all patients, L-ornithine was positively and significantly correlated with L-proline (p < 0.05, p < 0.001, p < 0.01, p < 0.0001, p < 0.0001 respectively). Only in patients 8, 14 and 15 was L-proline significantly correlated with two other members of the urea cycle, L-argininosuccinate and L-arginine (Supplementary Table 11). From these findings, proline metabolism and associated metabolic pathways appear important to HGG growth. This is supported by the expression of canonical proline pathway associated genes pyrroline-5-carboxylate reductase 1 (PYCR1) and ALDH18A1 (encoding P5CS protein) being expressed at higher levels in published GBM transcriptomic datasets (n = 418) relative to normal brain (n = 216) (Fig. 6B–C). A summary of metabolites of differential abundance between non-invasive and invasive regions taken into count all five patients and all intra-tumour regions, is provided in Supplementary Table 12. However, since the LC-MS analysis in our study represents a single time-point, it is not possible to determine whether increased synthesis or reduced degradation of L-proline is the contributing factor, thus requiring further study.

### 3.7 Regional heterogeneity in metabolism-associated gene expression

**Fig. 7:**
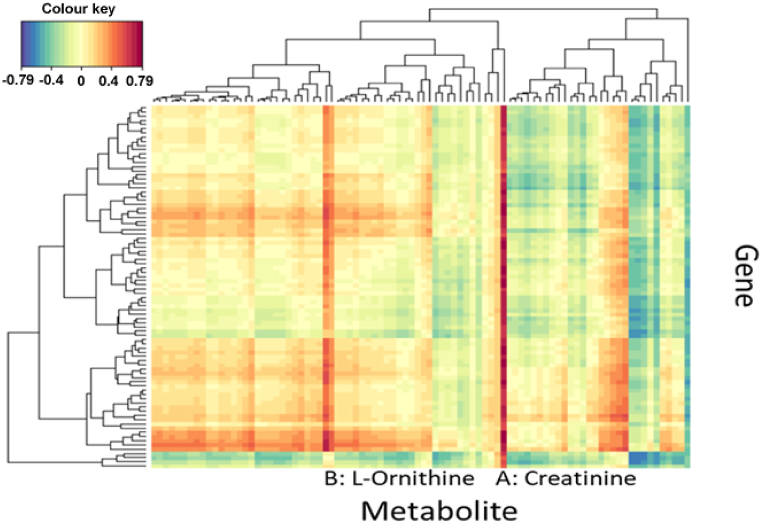
**Canonical correlation analysis identifying gene-metabolite associations.** Canonical correlation analysis between upregulated genes in the invasive margin and differentially abundant metabolites between the non-invasive and invasive regions. The positions of creatinine (A) and L-ornithine (B) with the strongest positive correlations are identified within the figure.

To determine if the basis of metabolic heterogeneity was due to an underlying heterogeneous transcriptomic profile, a multilevel model was applied to identify genes that were upregulated in the invasive margin, as these transcripts were hypothesised to be the most likely to influence the metabolomic profile displayed by this region. Only a few metabolism-related genes were identified (*LRP2*, *PDK4*) (Supplementary Table 13), indicating that metabolic transcripts were largely uniform between non-invasive and invasive regions. It is also possible that other upregulated genes have an as yet unidentified role in tumour metabolism, either directly or indirectly. Since only a few metabolism-related genes were identified as differentially expressed between invasive and non-invasive regions, the expression of genes encoding enzymes within glycolysis, PPP, TCA cycle, glutamine, serine/one-carbon, and lipid metabolism were examined to determine evidence of regionally heterogeneous expression. Most genes demonstrated minimal variation (maximum fold change of ∼2) across regions in patients 9 (Supplementary Fig. 6), 14 (Supplementary Fig. 7), and 15 (Supplementary Fig. 8). Interestingly, in all three patients the expression of *PDK4* (Supplementary Fig. 6A), Supplementary Fig. 7A and Supplementary Fig. 8A) and *LRP2* (Supplementary Fig. 6G, Supplementary Fig. 7G and Supplementary Fig. 8G) were higher in the invasive region (R5) compared to non-invasive regions, confirming the combined analyses presented in Supplementary Table 12. These findings indicate that intratumour heterogeneity in GBM does not extend to most metabolism-related genes, possibly accounting for the relative homogeneity observed for some metabolites across all regions. Integration of the upregulated genes in the invasive margin with metabolomic data through canonical correlation analysis identified strong correlation scores for creatinine and L-ornithine (Fig. 7). However, the biological significance of this remains to be elucidated through *in vitro* functional models.

## 4 Discussion

The subclonal nature of GBM has been demonstrated through a range of techniques, including genome-wide copy number analysis, single-cell transcriptomics, and protein-level expression of RTKs [2,4,6]. Given the intimate association between gene expression and metabolism, it is of scientific interest to determine whether the genetic heterogeneity observed within GBM manifests phenotypically as a patchwork landscape of metabolically distinct niches, however inter-convertible or transient. Genetic heterogeneity often occurs within redundant signalling pathways that converge to regulate metabolic pathways required to generate energy and biomass for sustained cancer cell proliferation [35]. Metabolic heterogeneity within gliomas has been shown across tumour grades [36], but has only been investigated in the context of a single tumour in cancers of the lung [15] and kidney [37]. We performed multi-region sampling in five HGG patients to profile intratumour metabolic heterogeneity following successful implementation of this technique in characterising genetic variation within a spatial context [4].LC-MS based metabolomic and lipidomic profiles obtained from four to five tumour regions per patient showed varied extent of metabolic heterogeneity between patients and within an individual. Most patients demonstrated heterogeneity at the metabolomic level, although several patients (particularly patients 9, 14, and 15) featured overlapping metabolomic profiles derived from non-invasive regions, indicating similarities in terms of overall metabolism. However, it was evident in all patients that the metabolomic profile within the invasive margin was distinct compared to that observed for the non-invasive regions. Consistently higher levels of L-proline within non-invasive intra-tumour regions relative to the GBM invasive margin in all five patients, suggests that increased synthesis or reduced catabolism of L-proline is important to tumour growth. Increased abundance of L-proline co-occurred with a similar increase in its catabolic breakdown product, 1-pyrroline-5-carboxylate (P5C). Indeed, the pattern finder feature of MetaboAnalyst indicated that L-proline and P5C were strongly correlated with a highly significant (p < 0.01) score of 0.8 and above, across all patients. Heterogeneity in canonical metabolites belonging to well described pathways, including glycolysis, TCA cycle, energetics, and amino acid metabolism was observed in all patients but to different extents: patient 6 (18 out of 42 significantly variant by ANOVA), patient 8 (39/42), patient 9 (10/42), patient 14 (39/42), and patient 15 (29/42). Only the canonical metabolites D-glucose and L-ornithine demonstrated significant regional heterogeneity across all patients, indicating that intratumour metabolic heterogeneity is patient-specific.

The extent of heterogeneity varied between patients and at the individual level. For instance, several metabolites within the glycolysis pathway showed regional variation in patient 6, in contrast to the relatively invariant profile in patient 15. Overlap of metabolomic profiles from different regions provided evidence of metabolic niches. The existence of metabolic niches may be related to the ability of the tumour vasculature to perfuse the oncogenic mass, as has been demonstrated through combined dynamic contrast-enhanced MRI (DCE-MRI) and ^13^C-isotopic analysis of lung cancer metabolic heterogeneity [15]. The relatively homogenous metabolomic profile of *IDH1* mutant patient 15 is of note since genetic lesions within *IDH1* affect both gene expression and metabolism [38,39]. With a larger cohort, it can be investigated if mutant *IDH1* expression results in a more metabolically homogenous tumour given the strength of this oncogenic lesion in driving tumourigenesis and metabolic reprogramming, in contrast to *IDH1* wildtype GBMs that are characterised by several oncogenic lesions, including chromosome7 loss/chromosome 10 gain, *CDKN2A* deletion, and amplification of *EGFR* and *PDGFRA* [40]. However, *IDH1* mutations have been demonstrated to be subclonal in 20 % of GBMs [41], levels of amino acids, glutathione metabolites, choline derivatives, and TCA cycle intermediates were altered in mutant *IDH1*- and *IDH2*-expressing cells, and mutated IDH1-R132H enzyme can dominant-negatively inhibit IDH1-WT isocitrate dehydrogenase activity, meaning that the extent of metabolic heterogeneity may be influenced by the dominance of *IDH1* mutations within the tumour hierarchy. Furthermore, whilst oncogenic process associated with IDH-based metabolic heterogeneity/homogeneity may be sufficient to promote GBM primary tumour progression, there remains a knowledge gap as to whether such associations are necessary for re-growth of GBM from post-treatment residual disease cells from the GBM invasive margin and brain parenchyma beyond.

Complicating the phenomenon of genetically induced metabolic re-programming is the capacity of cancer cells to undergo metabolic plasticity within a low nutrient or oxygen tumour microenvironment. Kucharzewska and colleagues highlighted several metabolic changes in GBM cells cultured in hypoxic conditions [42]. Stochastic determination of metabolic phenotypes is also observed since isogenic glioma stem-like cells (GSCs) can adopt either glycolysis or utilise oxidative phosphorylation to generate energy. In each GSC subtype, hypoxic stress increased the expression of glycolysis-related genes and promoted lactate production [43].This is understandable because under anaerobic conditions the cells rewire their metabolism with elevated glycolysis and lactate production fluxes to meet the need of ATP and regenerate NAD+. Patient 6 demonstrated evidence of a glycolytic switch in non-invasive regions compared to the invasive margin. Furthermore, heterogeneity in relative abundance of glycolytic metabolites, including G3P and pyruvate within intra-tumour regions, was observed. Hypoxia has been demonstrated to reduce pentose phosphate pathway activity and upregulate glycolytic enzymes leading to a “go” (i.e., migratory) instead of “grow” (i.e., proliferative) phenotype [44,45]. However, the expression of several glycolytic and PPP enzymes was largely homogenous across all regions, conflicting with hypotheses based on current understanding of associations between metabolism and invasion/proliferation.

Heterogeneity was also observed in TCA cycle metabolites that may reflect dynamic flux changes within connected metabolic pathways. Citrate, which is consumed in *de novo* fatty acid synthesis [46], was present at lower levels in the non-invasive regions of patients 6 and 15. Hypoxia induces reductive metabolism of glutamine to generate citrate for lipid synthesis via wildtype and mutant IDH1 [47,48]. Evidence of this process within patient 6 and 15 was not immediately obvious since glutamine and isocitrate levels were relatively uniform and citrate pools were reduced within non-invasive regions. Collectively, the findings from patient 15 hint at the lack of a hypoxia-induced metabolic phenotype, supporting the observations of Kickingereder and colleagues of strong inhibition of *HIF1A* and decreased expression of HIF1α target genes in *IDH1* mutant gliomas [49]. Patient 15 also featured glucose levels that were only moderately reduced and lower lactate levels in non-invasive regions compared to the invasive margin, both of which do not strongly support induction of a glycolytic phenotype due to the development of hypoxia.

Santandreu and colleagues provided evidence of intratumour metabolic heterogeneity in mitochondria following identification of a higher respiratory rate and fewer antioxidant systems within the periphery compared to the centre of the tumour [50]. This phenotypic spectrum is likely influenced by the development of hypoxia, often within the centre of solid cancers, which increases ROS production [51]. Glycolytic regions within patient 6 showed increased levels of oxidised glutathione. Patients 6 and 15 also demonstrated increased levels of most essential, conditionally essential (in context of illness and stress, e.g., arginine, cysteine, glutamine, tyrosine, glycine, proline, and serine), and non-essential amino acids within non-invasive regions compared to the invasive margin. Of therapeutic interest was the increased concentrations of L-proline and its strong correlation with its immediate breakdown product P5C in non-invasive regions. Proline biosynthesis is documented to support redox homeostasis in IDH1-mutant glioma by reducing the NADH/NAD + ratio via PYCR1 activity, which enables continuation of TCA cycle activity when flux through the electron transport chain is limiting as seen in hypoxic conditions [52]. In line with this, Liu and colleagues showed that cycling between the two metabolites recycles NAD + units for use in glycolysis and the pentose phosphate pathway [53]. In contrast, proline catabolism via *PRODH* was shown to support spheroidal breast cancer growth via ATP production and was also found to be increased in lung metastases compared to primary breast cancer tumours using ^13^C6-glucose as a tracer [54]. GBM cells that have infiltrated normal brain show increased gene expression of *PRODH* compared to tumour cells derived from the core, calling for further study into the role of proline in tumour metabolism [55].

We attempted to integrate transcriptomic and metabolomic data to determine associations between genetic and metabolic heterogeneity. However, genes that were upregulated between the invasive and non-invasive regions were largely associated with neuronal myelin sheath processes, (e.g., myelin associated glycoprotein (*mag*), myelin associated oligodendrocyte basic protein (*mobp*), myelin oligodendrocyte glycoprotein (*mog*), and brain enriched myelin associated protein 1 (*bcas1*; Supplementary Table 13), and therefore did not provide useful information pertaining to gene-metabolite interactions within a spatial context. Similarly, a lack of enrichment for metabolic processes was observed in the multi-region transcriptomics analysis conducted by Sottoriva and colleagues [4].

Overall, there are two therapeutic implications with regards to the identification of metabolic heterogeneity: 1) significantly varying metabolic substrates (e.g. glucose, serine, glycine, etc) may represent nutrients that are limiting for tumour growth due to non-oncogene addictions; therefore, further reduction through pharmacological means may facilitate tumour growth reduction. 2) metabolites that are minimally variant across regions and patients (e.g. glutamine) may be amenable for therapeutic targeting in the majority of HGG patients, irrespective of genetic subgrouping. Both hypotheses can be tested in larger cohorts, which was a limitation of our study. There is indeed precedence for efficacious disrupting of pyrimidine pools in high-grade glioma via arginine depletion [56].

In a larger cohort of patients, the identification of consistent metabolomic alterations can be investigated as biomarkers of invasion alongside histopathological and MRI data and correlated with survival to identify metabolic factors associated with prognosis. Other limitations to the study are inherent to the chosen methodology. LC-MS analysis only provides identification of metabolites and their relative abundances. In contrast, LC-MS analysis in combination with stable isotope (e.g., 13C) labelling can be used for metabolic flux analyses. ^13^C-isotope-labelled substrates have been implemented to great effect to study substrate utilisation and the destination of carbon units, as shown by the usage of 1,2-^13^C-acetate in GBM and brain metastases [57]. Irrespective of this, it is imperative that variations in metabolite levels such as that shown by this study, are established to vary significantly beyond normal brain physiological levels Moreover, the presence of a normal brain component within the invasive margin precludes the identification of metabolic markers for invading cells. The results drawn using the chosen methodology of analysing multi-region sampled tumour fragments through LC-MS are further confounded by the lack of data delineating the contribution of non-tumour or stromal cells, including immune cells, neurons, and endothelial cells, to the metabolomes within the non-invasive and invasive regions. Future attempts to fully characterise intratumour metabolic heterogeneity should also utilise single-cell isolation strategies in conjunction with developments within the metabolomic and lipidomic fields in relation to single-cell applications. To overcome this confounding issue, our future work will adapt a methodological pipeline which we developed to specifically isolate infiltrative GBM subpopulation(s) based on fluorescence-activated cell sorting (FACS) of 5ALA fluorescing surgically resected tissue [58]. We showed that the infiltrative tumour margin, where GBM cells penetrate healthy brain parenchyma, harbours distinct transcriptomic profiles compared to the tumour core and where the GBM infiltrative margin gene signature is more similar to GBM recurrent tumours, than to the core region of the primary tumour [59]. Whilst the process of enzymatic dissociation of surgical biopsies followed by FACS did not confound downstream RNA-sequencing analyses (i.e., only a subset of stress-related genes was altered), this process will likely compound metabolic profiling. Therefore, we are currently optimising methods for metabolite and lipid extraction directly, and solely, from the GBM faction of infiltrative margin tissue. If this is feasible, we plan to conduct LC-MS-based metabolomic profiling of a pure population(s) of infiltrative GBM using a larger prospective cohort of patients.

## 5 Conclusions

This study demonstrates the applicability of LC-MS as an analytical method for metabolomic profiling of intratumour surgical biopsies from patients undergoing GBM resective surgery. Collectively, our findings reveal that the clinically relevant GBM invasive margin exhibits a distinct metabolome and lipidome relative to non-invasive intratumour regions. Nevertheless, the predominance of neuronal transcriptomic signatures in the invasive margin indicates the challenge to discriminate tumour-specific metabolomic signatures in this cellular region. 5ALA fluorescence-based separation of tumour from astrocytic/immune fractions, and subsequent metabolite extraction, presents a feasible approach to overcome this challenge. Furthermore, the small sample size in our study cautions against generalisability of findings with regards to specific differences in metabolic species. Extending our study to a larger cohort of patients, will provide greater confidence in the identification of metabolomic alterations as putative biomarkers of invasion should be complemented by histopathological and MRI information. Such data could be correlated to survival to identify metabolites/metabolic pathways which may predict time to tumour recurrence, but also elucidate metabolic processes which are amenable for therapeutic targeting of post-residual disease, prior to manifestation of radiologically confirmed recurrence.

## CRediT authorship contribution statement

**James Wood:** Writing – original draft, Methodology, Data curation, Conceptualization. **Stuart J. Smith:** Conceptualization. **Marcos Castellanos-Uribe:** Methodology, Data curation. **Anbarasu Lourdusamy:** Methodology. **Sean T. May:** Writing – review & editing. **David A. Barrett:** Writing – review & editing. **Richard G. Grundy:** Supervision. **Dong-Hyun Kim:** Writing – review & editing, Data curation. **Ruman Rahman:** Writing – review & editing, Writing – original draft, Supervision, Conceptualization.

## Data availability statement

The original contributions presented in this study are included in the article, further inquiries can be directed to the corresponding author. Gene expression microarray data is available via ArrayExpress (accession number: E-MTAB-14600; study title: ‘Glioblastoma intra-tumour transcriptomics’).

## Declaration of Competing Interest

The authors declare that they have no known competing financial interests or personal relationships that could have appeared to influence the work reported in this paper.
