# Supplementary material for: Metabolomic characterisation of the glioblastoma invasive margin reveals a region-specific signature
Source: Heliyon. 2024 Dec 21;11(1):e41309. doi: 10.1016/j.heliyon.2024.e41309 (PMC11732679; doi:10.1016/j.heliyon.2024.e41309)
Supplement: Multimedia component 2 [file mmc2.docx]

**
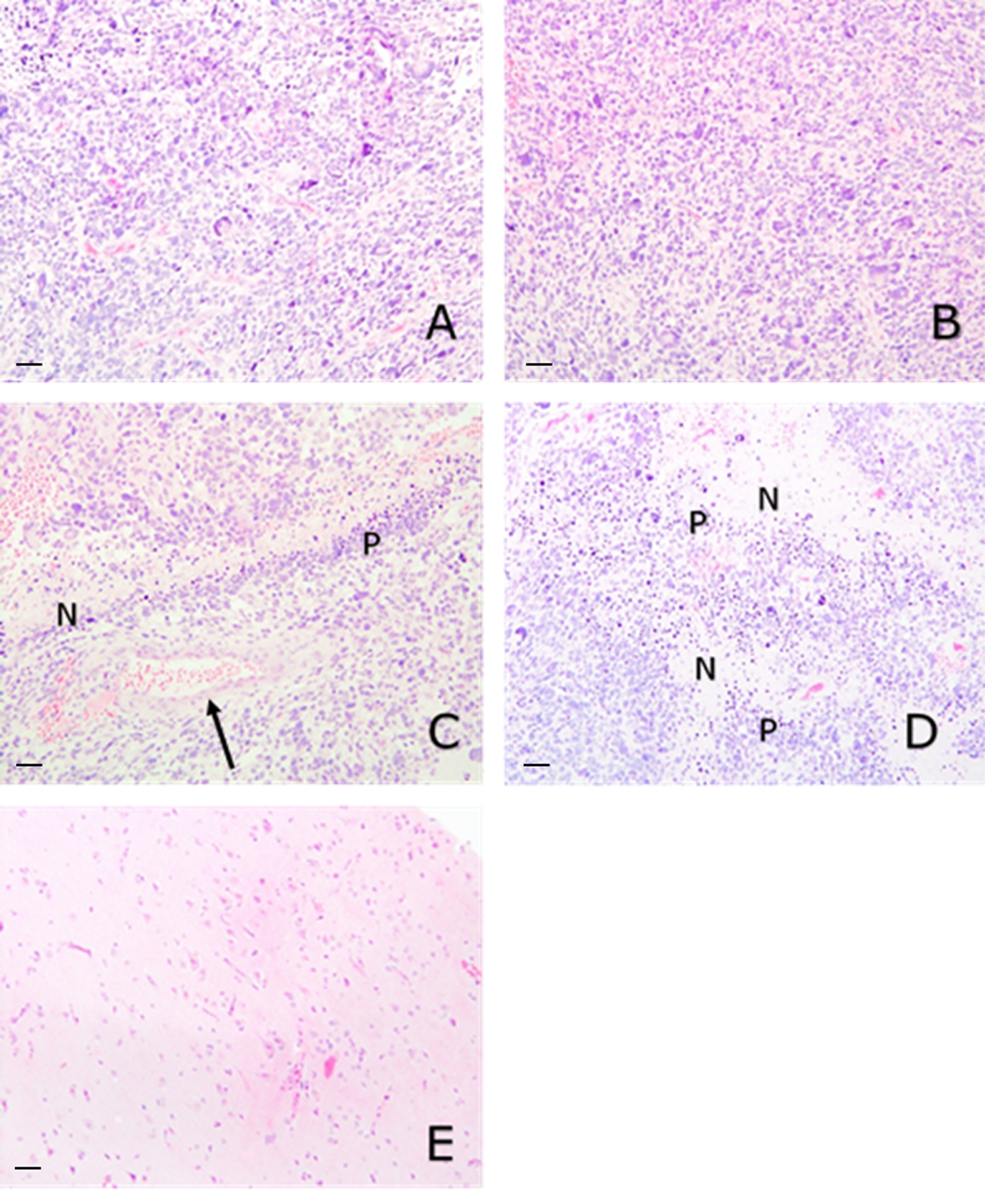
**

**Supplementary Figure 1: Representative example of haematoxylin and eosin staining of tumour fragments sampled from patient 15.** (A) Tissue sampled from the lateral temporal region demonstrates high cellularity, anaplasia, nuclear pleomorphism, and a mixture of dark nuclei suggesting hyperchromatism. (B) Anterior region tissue exhibits features of anaplasia, nuclear pleomorphism and hyperchromatism. (C) Tissue from the core region shows the highest cell density and evidence of mitoses. An area of necrosis (N) is observed and is lined with pseudo palisading cells (P). A blood vessel can an also be seen (arrow), reflecting the highly vascular tumour core. (D) Posterior temporal region tissue contains a large section of necrotic tissue (N) and palisading cells (P), as well as features indicative of hyperchromatism. (E) The invasive margin has a low nuclear to cytoplasmic ratio and the tissue is not as anaplastic in comparison to other regions. Brightfield images were taken at 10x magnification. Scale bars represent 100 µm in length.

**
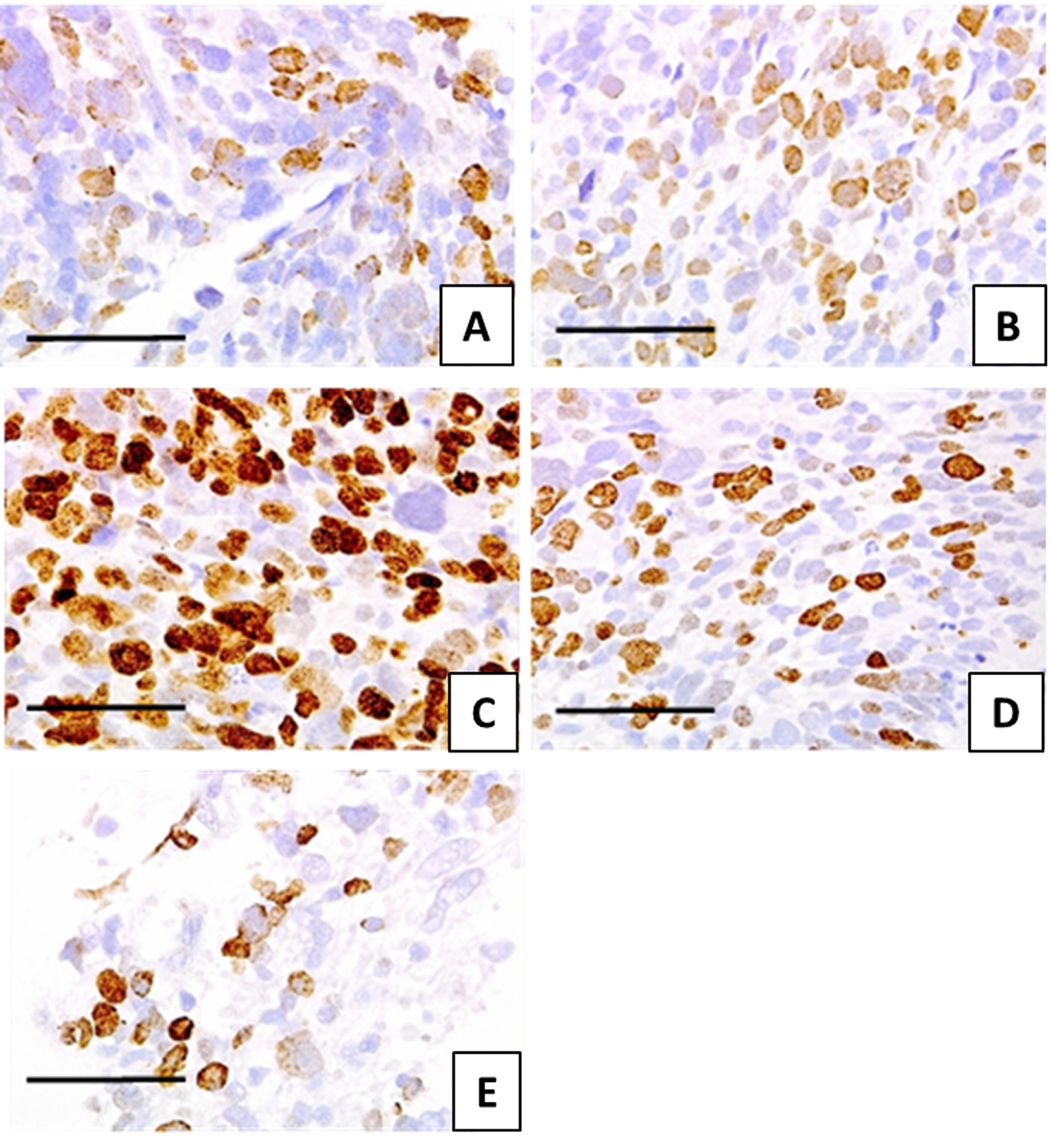
**

**Supplementary Figure 2: Representative example of a Ki67 immunohistochemical staining of tumour fragments sampled from patient 15.** (A, B and D) Immunohistochemistry was performed using Ki-67 at a dilution of 1:50. Almost a third of cells are stained positive for Ki67 in the lateral temporal, anterior and posterior temporal regions. (C) Strong staining within the central core indicates that this is the most proliferative region. (E) The invasive margin is the least proliferative with minimal staining of cells. Tumour images were taken at x40 magnification. Scale bars represent 10μm in length.

#
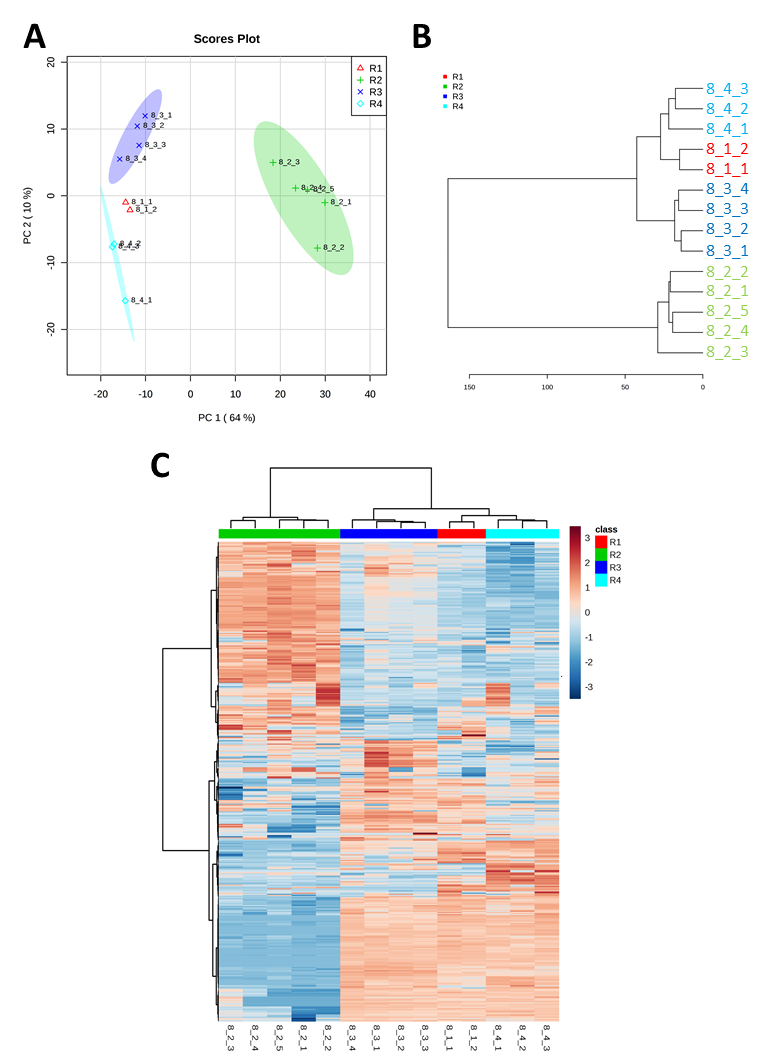


**Supplementary Figure 3:** **Metabolomics plots for patient 8**. (A) Dimensional reduction through PCA and visualisation of sample variation. (B) Hierarchical clustering analysis measuring dissimilarity between samples. (C) Heatmap overview of the metabolome. Sample IDs delineate the patient, region (symbol R), and replicate numbers, respectively.

**
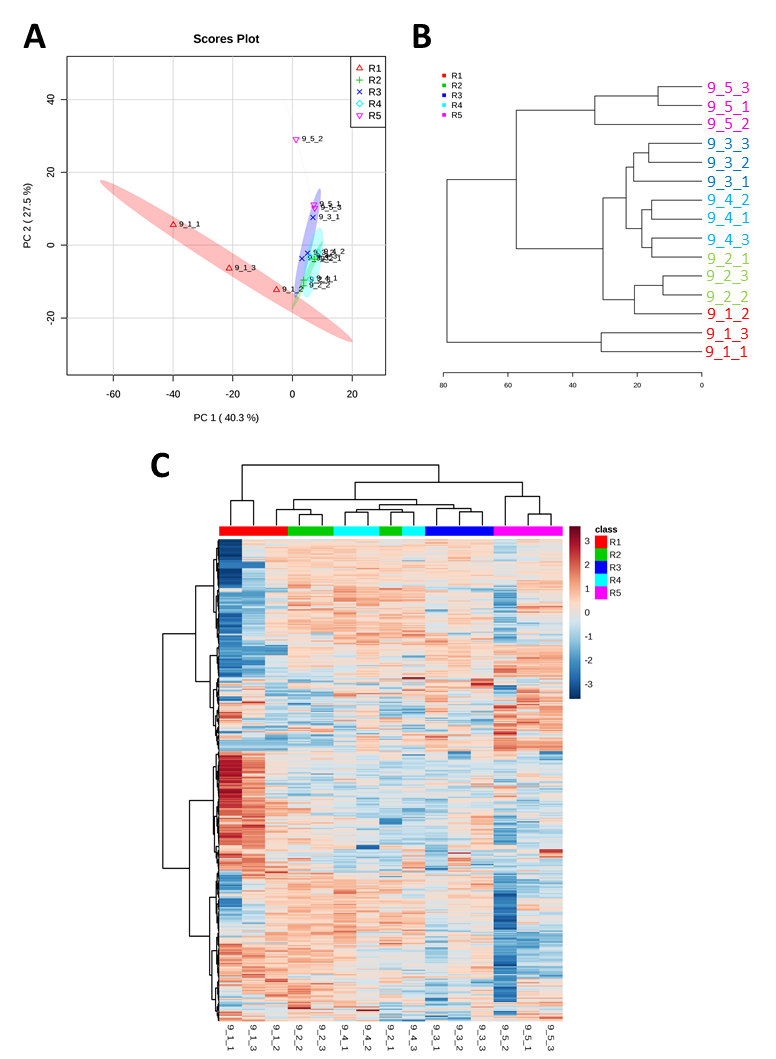
**

**Supplementary Figure 4: Metabolomics plots for patient 9.** (A) Dimensional reduction through PCA and visualisation of sample variation. (B) Hierarchical clustering analysis measuring dissimilarity between samples. (C) Heatmap overview of the metabolome. Sample IDs delineate the patient, region (symbol R), and replicate numbers, respectively.

**
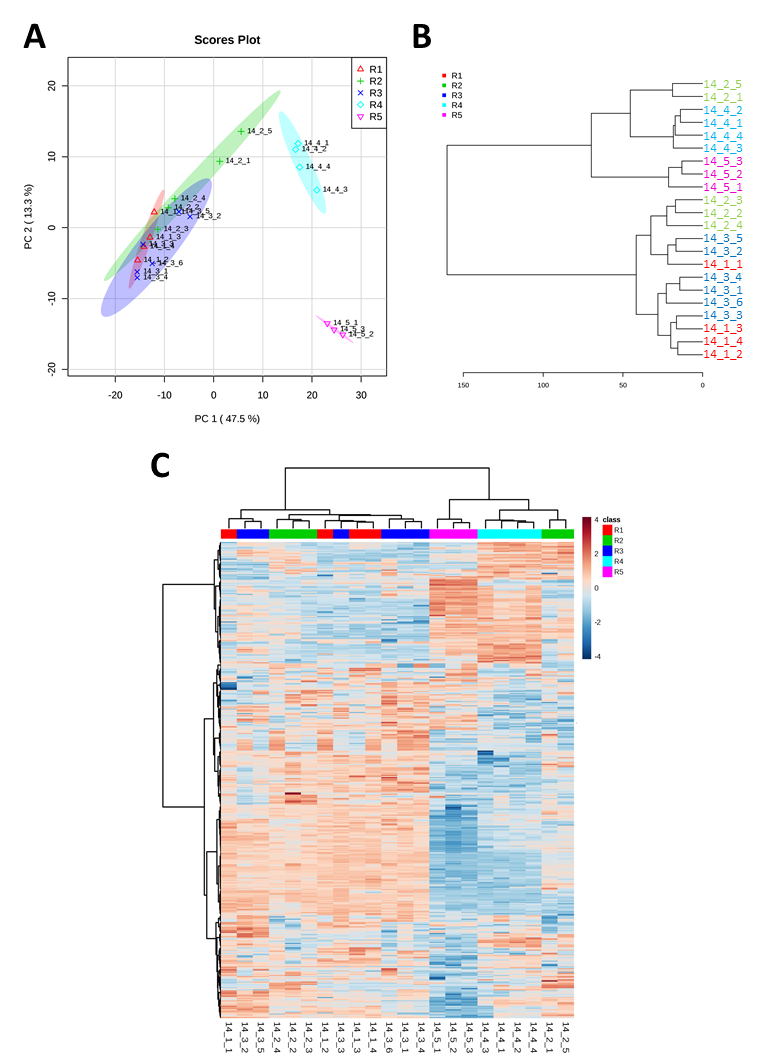
**

**Supplementary Figure 5: Metabolomics plots for patient 14**. (A) Dimensional reduction through PCA and visualisation of sample variation. (B) Hierarchical clustering analysis measuring dissimilarity between samples. (C) Heatmap overview of the metabolome. Sample IDs delineate the patient, region (symbol R), and replicate numbers, respectively.

**
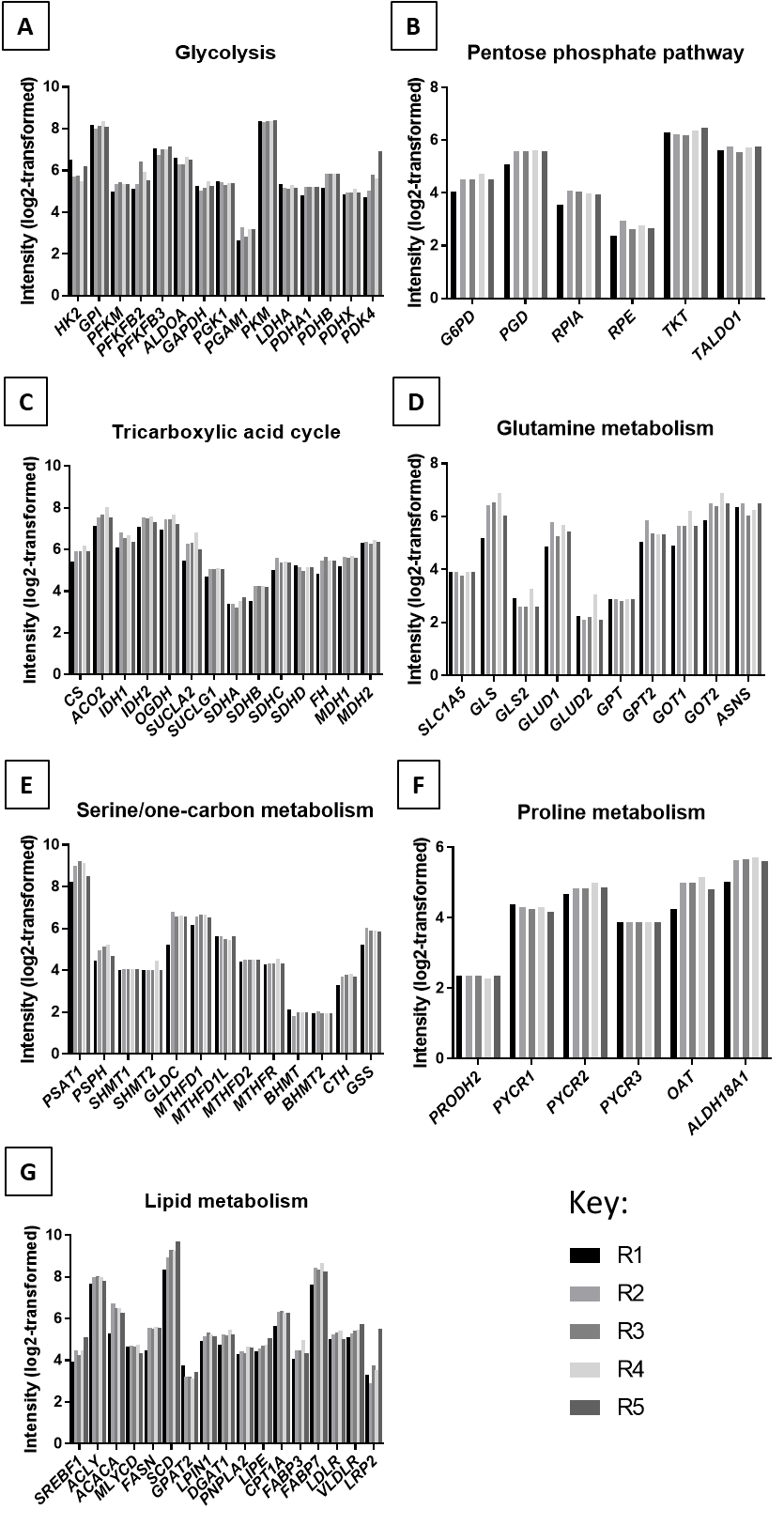
**

**Supplementary Figure 6: Regional expression of metabolism-related genes in patient 9.** The expression of genes encoding enzymes within the metabolic pathways in A-G are displayed as log2-transformed intensity values. Variation across regions (symbol R) are shown for patient 9.

**
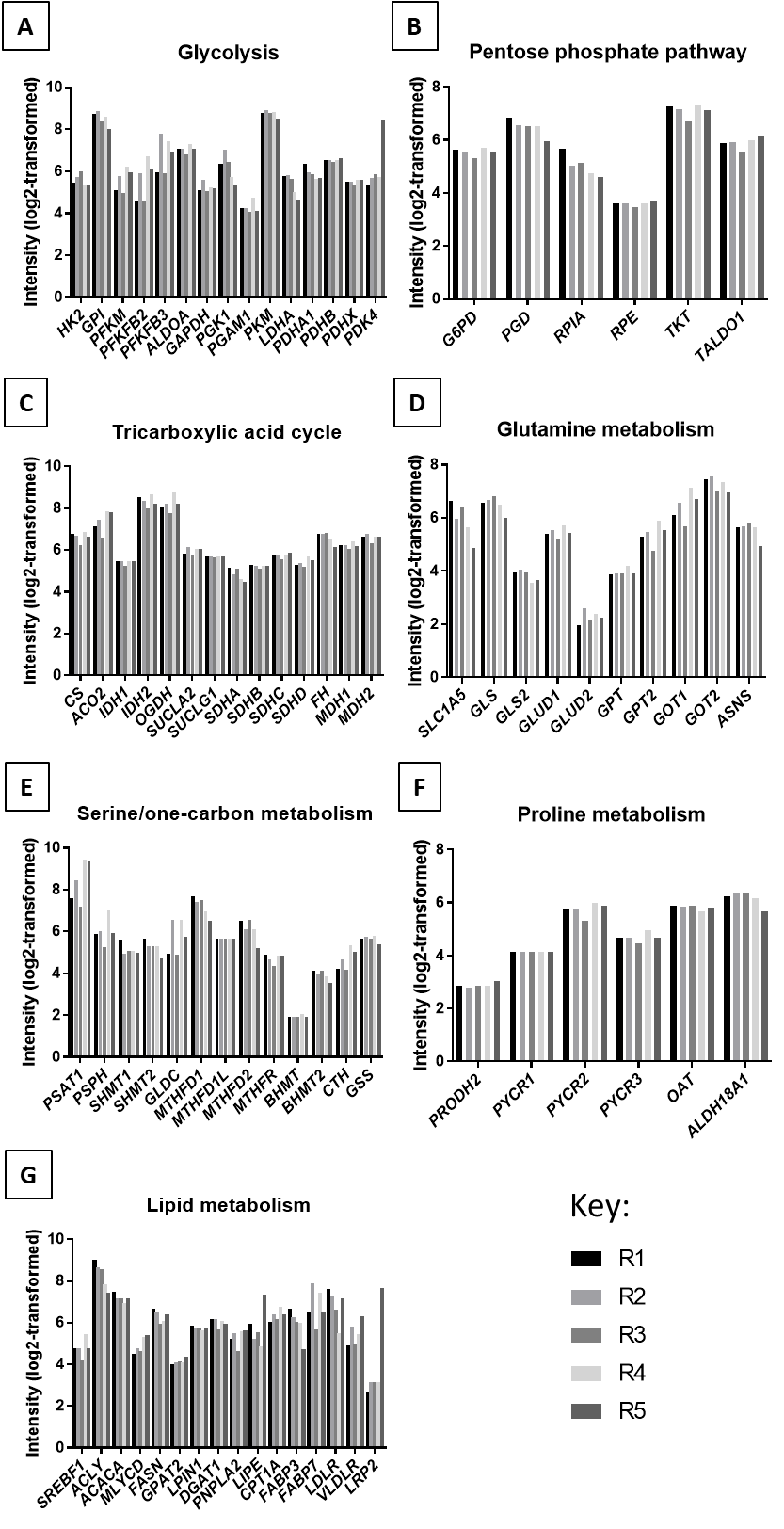
**

**Supplementary Figure 7: Regional expression of metabolism-related genes in patient 14.** The expression of genes encoding enzymes within the metabolic pathways in A-G are displayed as log2-transformed intensity values. Variation across regions (symbol R) are shown for patient 14.

**
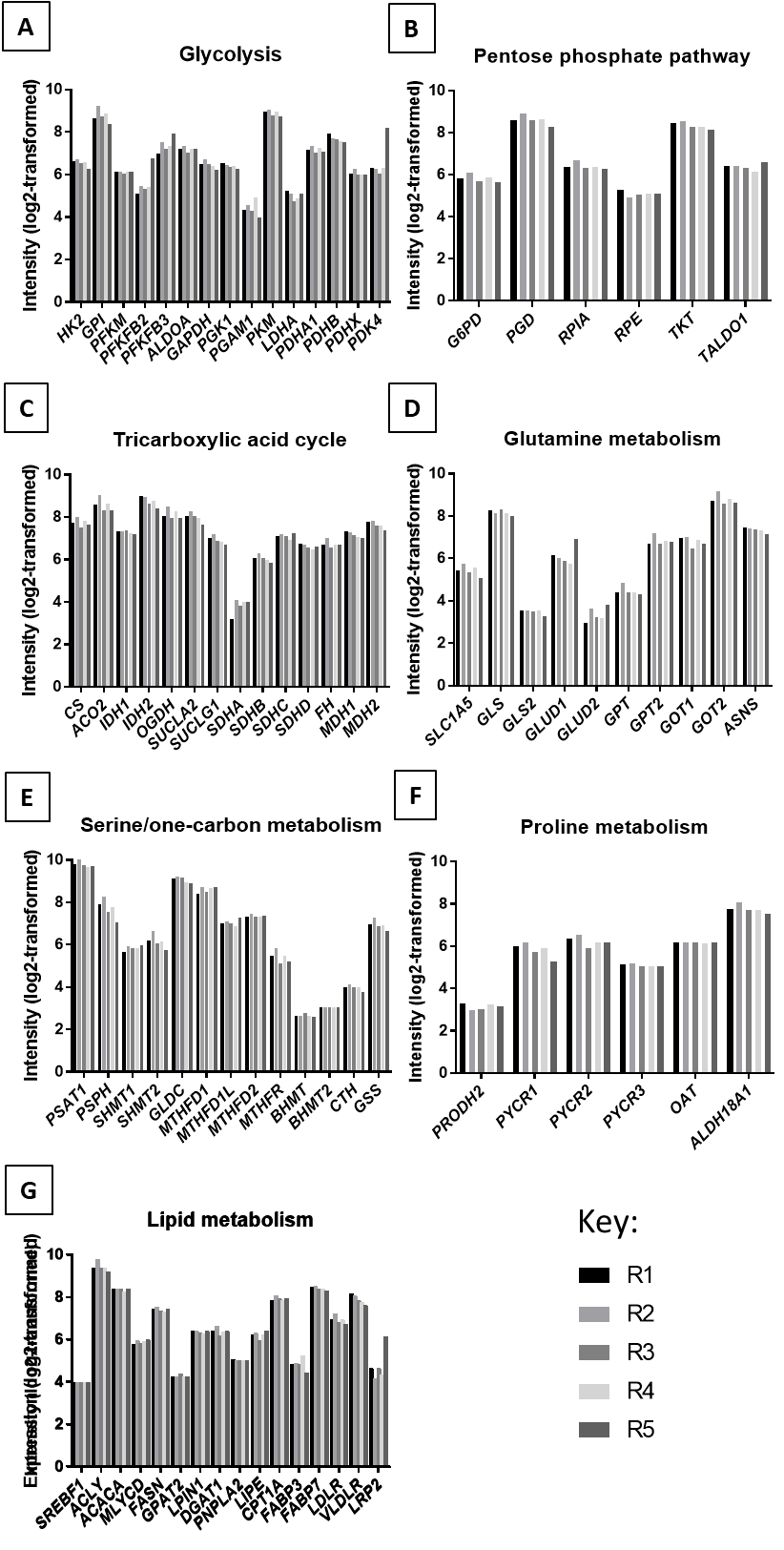
**

**Supplementary Figure 8: Regional expression of metabolism-related genes in patient 15.** The expression of genes encoding enzymes within the metabolic pathways in A-G are displayed as log2-transformed intensity values. Variation across regions (symbol R) are shown for patient 15.

**Supplementary Table 1: Clinical information for the five patients chosen for intra-tumour metabolomics.** Details are provided about the histological diagnosis (GBM: glioblastoma multiforme; MGNT: malignant glioneuronal tumour), *IDH1* status (WT: wildtype), 5-amino-levulinic acid (5-ALA) administration, radiotherapy (RT) in Gray units, chemotherapy (CT), and response to temozolomide (TMZ). 5-aminolevulinic acid (5ALA) was administered orally to patients 2-4 hours prior to surgery at 20mg/kg dose. All patients underwent craniotomy with intra-operative image guidance and visualization of 5ALA induced fluorescence using an appropriately equipped operative microscope (Leica OH-X). Multi-region sampling was conducted as per Smith SJ et al. *Neurooncology Advances* 2(1):vdaa087 (2020), and sample location determined using image guidance. Samples were taken from non-fluorescent or minimally fluorescent tumor core (corresponding to T1 with gadolinium non-enhancing or heterogeneously enhancing central tumor), viable fluorescent tumor rim (corresponding to peripheral strongly gadolinium enhanced areas on T1 MRI) and from the areas of 5ALA induced fluorescence furthest into the area of MRI T2 high signal beyond the bulk tumor, where tumor blended into brain in an invasive fashion (non-enhancing on T1 with gadolinium). Histological diagnosis of glioblastoma was confirmed by intra-operative smear and formal post-operative diagnosis (including IDH mutations, ATRX mutation and MGMT methylation status) by the regional brain tumor neuropathology service.

**Supplementary Table 2: Sample information for the five patients chosen for the intra-tumour metabolomics.**

**Supplementary Table 3: Metabolic variation across region and region types in patient 6.** Metabolites highlighted in red showed RSD values >30% in QC samples. ID confidence scores delineate the confidence of the putative metabolite identification, where 10 = standard retention time within 5%, in preferred database (Human Metabolome Database), 8 = standard retention time within 5%, in preferred database and related peak (mz match). Fold changes are invasive to non-invasive comparisons and are displayed as logarithms to the base 2 (log2FC). Fold changes >0.58 or < -0.58 are depicted in red and green, respectively. Statistical evaluation of variation between regions and region types was performed using a one-way ANOVA model and two-sample *t*-test, respectively. Significance scores were corrected for multiple comparisons using false discovery rate (FDR). ns = not significant; * = p<0.05; ** = p<0.01; *** = p<0.001; **** = p<0.0001.

**Supplementary Table 4: Metabolic variation across region and region types in patient 15.** Metabolites highlighted in red showed RSD values >30% in QC samples. ID confidence scores delineate the confidence of the putative metabolite identification, where 10 = standard retention time within 5%, in preferred database (Human Metabolome Database), 8 = standard retention time within 5%, in preferred database and related peak (mz match). Fold changes are invasive to non-invasive comparisons and are displayed as logarithms to the base 2 (log2FC). Fold changes >0.58 or < -0.58 are depicted in red and green, respectively. Statistical evaluation of variation between regions and region types was performed using a one-way ANOVA model and two-sample *t*-test, respectively. Significance scores were corrected for multiple comparisons using false discovery rate (FDR). ns = not significant; * = p<0.05; ** = p<0.01; *** = p<0.001; **** = p<0.0001.

**Supplementary Table 5: Metabolic variation across region and region types in patient 8.** Metabolites highlighted in red showed RSD values >30% in QC samples. ID confidence scores delineate the confidence of the putative metabolite identification, where 10 = standard retention time within 5%, in preferred database (Human Metabolome Database), 8 = standard retention time within 5%, in preferred database and related peak (mz match). Fold changes are invasive to non-invasive comparisons and are displayed as logarithms to the base 2 (log2FC). Fold changes >0.58 or < -0.58 are depicted in red and green, respectively. Statistical evaluation of variation between regions and region types was performed using a one-way ANOVA model and two-sample t-test, respectively. Significance scores were corrected for multiple comparisons using false discovery rate (FDR). ns = not significant; * = p<0.05; ** = p<0.01; *** = p<0.001; **** = p<0.0001.

**Supplementary Table 6: Metabolic variation across region and region types in patient 9.** Metabolites highlighted in red showed RSD values >30% in QC samples. ID confidence scores delineate the confidence of the putative metabolite identification, where 10 = standard retention time within 5%, in preferred database (Human Metabolome Database), 8 = standard retention time within 5%, in preferred database and related peak (mz match). Fold changes are invasive to non-invasive comparisons and are displayed as logarithms to the base 2 (log2FC). Fold changes >0.58 or < -0.58 are depicted in red and green, respectively. Statistical evaluation of variation between regions and region types was performed using a one-way ANOVA model and two-sample *t*-test, respectively. Significance scores were corrected for multiple comparisons using false discovery rate (FDR). ns = not significant; * = p<0.05; ** = p<0.01; *** = p<0.001; **** = p<0.0001.

**Supplementary Table 7: Metabolic variation across region and region types in patient 14.** Metabolites highlighted in red showed RSD values >30% in QC samples. ID confidence scores delineate the confidence of the putative metabolite identification, where 10 = standard retention time within 5%, in preferred database (Human Metabolome Database), 8 = standard retention time within 5%, in preferred database and related peak (mz match). Fold changes are invasive to non-invasive comparisons and are displayed as logarithms to the base 2 (log2FC). Fold changes >0.58 or < -0.58 are depicted in red and green, respectively. Statistical evaluation of variation between regions and region types was performed using a one-way ANOVA model and two-sample t-test, respectively. Significance scores were corrected for multiple comparisons using false discovery rate (FDR). ns = not significant; * = p<0.05; ** = p<0.01; *** = p<0.001; **** = p<0.0001.


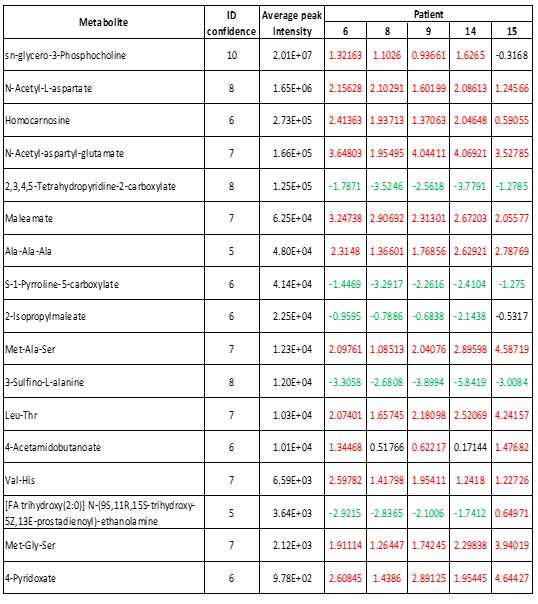


**Supplementary Table 8: List of metabolites commonly identified across all five patients as significantly variant between invasive and non-invasive regions using *t*-test.** Metabolites highlighted in red showed RSD values >30% in QC samples. ID confidence scores delineate the confidence of the putative metabolite identification, where 10 = standard retention time within 5%, in preferred database (Human Metabolome Database), 8 = standard retention time within 5%, in preferred database and related peak (mz match), 7 = standard retention time within 5%, not in preferred database and related peak (mz match), 6 = calculated retention time within 50%, in preferred database and related peak (mz match), 5 = calculated retention time within 50%, not in preferred database and related peak (mz match).

Fold changes in metabolite peak intensities between the invasive and non-invasive regions are displayed as logarithms to the base 2 (log2FC). Fold changes >0.58 or < -0.58 are depicted in red and green, respectively.

**Supplementary Table 9: Metabolite set enrichment analysis of metabolites identified as differentially abundant based on a multilevel linear model.** The table details the top five enriched metabolite sets based on significantly differentially abundant metabolites with log2 fold-change >0.58 or < -0.58. Significant raw *p*-values (Raw p) for enriched metabolite sets were adjusted for multiple comparisons using the Holm (Holm p) or false-discovery rate (FDR) method.

**Supplementary Table 10: Pathway analysis of metabolites identified as differentially abundant based on a multilevel linear model.** The table details the top five impacted metabolic pathways based on significantly differentially abundant metabolites with log2 fold-change >0.58 or < -0.58. Significant raw *p*-values (Raw p) for enriched metabolite sets were adjusted for multiple comparisons using the Holm (Holm p) or false-discovery rate (FDR) method. Impact scores range between 0 and 1.

**Supplementary Table 11: Regional comparison of metabolites associated with proline**

**metabolism and correlation with L-proline**. ID confidence scores delineate the confidence of the putative metabolite identification, where 10 = standard retention time within 5%, in preferred database (Human Metabolome Database), 8 = standard retention time within 5%, in preferred database and related peak (mz match), 6 = calculated retention time within 50%, in preferred database and related peak (mz match). Fold changes are displayed as logarithms to the base 2 (log2FC). Fold changes >0.58 or < -0.58 are depicted in red and green, respectively. Significance scores were FDR-corrected for multiple comparisons. ns = not significant; * = p<0.05; ** = p<0.01; *** = p<0.001; **** = p<0.0001.

**Supplementary Table 12: Metabolites of differential abundance between non-invasive and invasive regions using a multilevel linear model.**


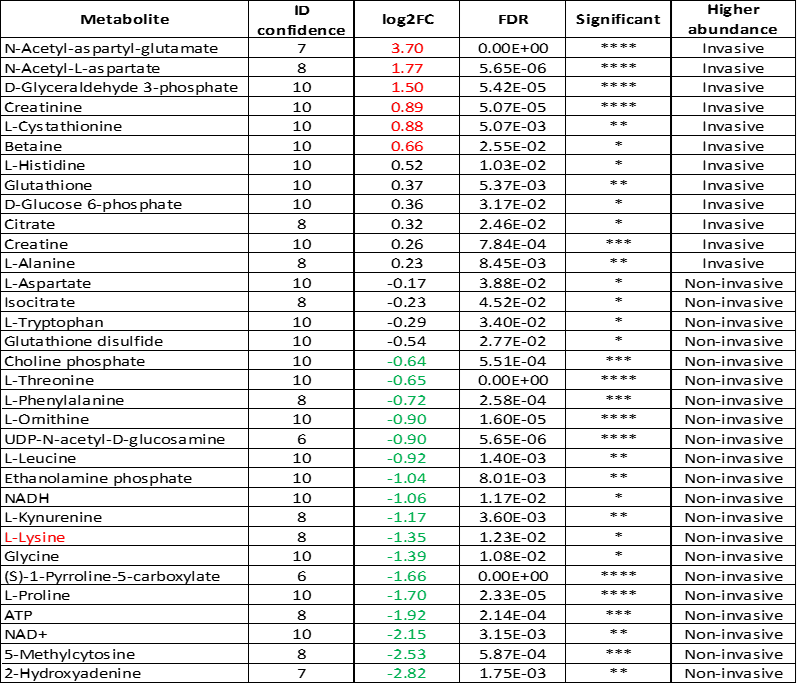


Metabolites highlighted in red showed RSD values >30% in QC samples. ID confidence scores delineate the confidence of the putative metabolite identification. Fold changes are displayed as logarithms to the base 2 (log2FC). Fold changes >0.58 or < -0.58 are depicted in red and green, respectively. The region type with the higher peak intensity is indicated. Significance scores were FDR-corrected for multiple comparisons. ns = not significant; * = p<0.05; ** = p<0.01; *** = p<0.001; **** = p<0.0001.

**
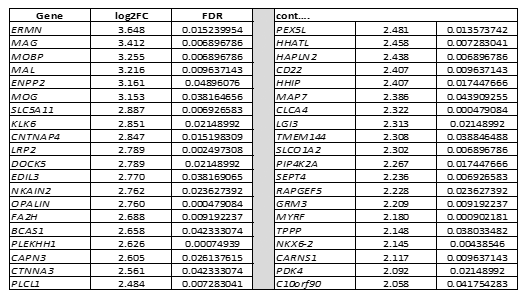
**

**Supplementary Table 13:** **Significantly upregulated genes within the invasive margin determined using a multilevel linear model.** The top 40 significantly upregulated genes within the invasive margin compared to the non-invasive regions are displayed. Myelin associated glycoprotein (*mag*), myelin associated oligodendrocyte basic protein (*mobp*), myelin oligodendrocyte glycoprotein (*mog*), and brain enriched myelin associated protein 1 (*bcas1*) were identified as gene s associated with neuronal myelin sheath processes exhibiting significantly upregulated gene expression in invasive margin relative to non-invasive margin. Genes are ordered according to fold changes to the logarithm of base 2 (log2FC). False discovery rate (FDR)-corrected *p*-values were calculated using a multilevel linear model.
